# The *jeong* and *haan* of Vincent van Gogh: neuropeptides of bondedness and loss

**DOI:** 10.3389/fpsyg.2024.1432175

**Published:** 2024-12-24

**Authors:** Sung W. Lee, Kathryn R. Cullen, Sung-ryun Rim, Carlee Toddes

**Affiliations:** ^1^Department of Bioethics and Medical Humanism, University of Arizona College of Medicine, Phoenix, AZ, United States; ^2^Department of Psychiatry and Behavioral Sciences, University of Minnesota Medical School, Minneapolis, MN, United States; ^3^Masonic Institute for the Developing Brain, University of Minnesota Medical School, Minneapolis, MN, United States; ^4^College of Liberal Arts, Pyeongtaek University, Pyeongtaek, Republic of Korea; ^5^Graduate School of Art Therapy, Pyeongtaek University, Pyeongtaek, Republic of Korea; ^6^Department of Neurobiology and Biophysics, University of Washington, Seattle, WA, United States

**Keywords:** pain, endorphin, attachment, loneliness, vasopressin, aggression, borderline personality disorder, euphoria

## Abstract

We introduce two Korean-named yet transcultural feelings, *jeong* and *haan*, to fill gaps in neuroscientific understanding of mammalian bondedness, loss, and aggression. *Jeong* is a visceral sense of connectedness to a person, place, or thing that may arise after proximity, yet does not require intimacy. The brain opioid theory of social attachment (BOTSA) supports the idea that *jeong* involves increased activity of enkephalins and beta-endorphins. We propose that withdrawal of *jeong*-related neuropeptides leads to original *haan*, a sense of “missingness” that is too subtle to be grossly dysphoric. Through narrative, cognitive appraisals, or moral assignments, however, original *haan* may transform into the feeling of constructed *haan—*resentment, bitterness, grievance, sorrow, or suppressed anger. In males, the transformation may be driven by arginine vasopressin, an ancient fight-or-flight neurohormone. Constructed *haan* may also be driven by vasopressin in females, though data is more sparse, and in both sexes it may depend on situational or societal context. Endogenous opioids inhibit vasopressin, so that when *jeong* diminishes, vasopressin release may become disinhibited. This relationship implies a companion to the BOTSA, which we articulate as the brain opioid and vasopressin theory of original and constructed *haan* (BOVTOCH). To illustrate, we reflect on borderline personality disorder, and Vincent van Gogh’s self-severing of his ear while living and working with Paul Gauguin, and fearing abandonment by him; yet to understand Van Gogh more completely we also present the brain opioid theory of stable euphoric creativity (BOTSEC), to model the subjective “highs” associated with creative flow states. Together these brain opioid theories may help to explain how feelings related to social bondedness can influence a range of phenomena. For example, opioid drug dependence may be, at least partly, a maladaptive response to feelings of isolation or disconnectedness; the health protective effects of social bonds could be related to tonic exposure to endogenous opioids and their anti-inflammatory properties; endogenous opioid-based social relational enhancement may contribute to placebo responding. Finally we conclude by pointing out the possibility of virtuous cycles of social connectedness and creativity, when feelings of bondedness and euphoric flow reinforce one another through endogenous opioid elevation.

## 1 Introduction

Throughout this long development, from 600 b.c. to the present day, philosophers have been divided into those who wished to tighten social bonds and those who wished to relax them.— Bertrand Russell, History of Western Philosophy (Russell, 2004).

Pain relief, social bondedness, and euphoria depend on a common neurobiological pathway. In this paper, we discuss the brain’s endogenous opioid system as a critical mechanism for all of these events. In doing so, we aim to accomplish three objectives:

First, we aim to introduce two Korean-named subtleties of feeling or awareness—*jeong* and *haan*, related to bondedness and loss, respectively—that may help expand the phenomenological repertoire of affective neuroscience.

Second, we aim to provide a brief overview of the opioid system and posit a novel schema for the role of natural opioidergic processes in modulating *jeong*, *haan* and creative flow states.

Third, to illustrate the overlapping neuropeptide mechanisms, we consider the subjective swings of euphoria and dysphoria felt by Vincent van Gogh, especially during the months and moments before and after he severed his ear in December 1888.

Along the way, we propose new theories for the conjoint influence of brain opioid decline and vasopressin elevation for motivating male aggression, and for elevated endogenous opioidergic tone for the subjectivity of creative flow. We will also contend that neuropeptide dynamics behind feelings of connectedness and loss can improve transdiagnostic insight into borderline personality disorder and self-injury, yet also relational behaviors seen in everyday life. Finally we will briefly discuss our theories as ways to consider the origins of the opioid epidemic, the influence of social connectedness on health, and the mechanisms of the placebo response.

Neuroscience has yet to produce a coherent account for the experiences of bondedness, loss, and creative flow, or a model that considers the implications of these feelings in flux. Aggression or self-harm driven by resentments or grievances has yet to disappear from any scale or type of human community, and here we articulate a neurobiological pathway from feelings of disconnectedness to a motivation to harm oneself or others. Moreover in our highly technology-driven world, attachment bonds are developing through (or to) human proxies including cell phones and generative AI-based chatbots, such that it is increasingly imperative that we understand the neurobiology involved in the transfer of our *Homo sapien* bonding or attachment mechanisms to socially representative objects. To help fill gaps of understanding relating to relatedness itself - some that exist now and others that may be forthcoming - this paper adduces phenomenology and language from non-Western culture and lexicon.

## 2 Does the science of attachment need to expand its phenomenology?

### 2.1 What we left behind in order to become WEIRD

In a seminal review paper (Henrich et al., 2010), the anthropologist Joseph Henrich and colleagues showed how a wide range of scientific inferences related to visual perception, moral reasoning, or concepts of fairness or cooperation, have been dependent on studies conducted on only a narrow slice of humanity. When researchers describe universal traits or patterns of *Homo sapien* feeling, thinking, or behavior, conclusions are typically being drawn from data collected from college students, often in the USA—whose minds were formed in Western, Educated, Industrialized, Rich, and Democratic, or WEIRD societies. And while the spread, reinforcement, and compounding of the Western mind’s outputs—including its characteristic individualism—has been a “secret of the success” of its civilization (Henrich, 2016), the work of Henrich and those in his wake has entailed a call for behavioral scientists to broaden their outlook and study samples (Masuda et al., 2020).

Subsequently, Henrich has proposed that this rapidly globalizing mentality emerged from an agenda of the early Catholic Church to weaken the power of *kinship* bonds (Henrich, 2020). For Christian teachings to spread, papal authorities put forth a “Marriage and Family Program” which included a limitation on cousin marriages, whose net effect, compounded over the centuries, was to create an impersonal sense of pro-sociality. This orientation eventually became the foundation for the axioms of modern philosophy, science, and jurisprudence, which place the highest priority on pure objectivity or impartiality, and which aim to remove the individual observer, with their unique interests and personal attachments, from the workings or evolution of society at large.

There have been shadows, though, that have come with the accomplishments of the WEIRD mind. Difficulty navigating mammalian kinship bonds left behind seems to be one of them, and the challenge even extends to our ability to discuss bondedness as a natural phenomenon. For example, consider this comment from the anthropologists Robin Dunbar and Susanne Shultz:

One reason why defining the nature of bondedness is so problematic seems to be that it is intrinsically an emotional process, and we have no adequate language with which to describe such relationships even for our own species. In this sense, the nature of our relationships is hidden even from our own capacities to comment on them (Dunbar and Shultz, 2010).

The origin of the problem, though, as Henrich has pointed out, seems to be at the level of history, not the species. Dunbar and Shultz go on to identify behaviorism, a paradigm which only accepts *observable* phenomena as objects of reality, as a cause for our limitation of understanding:

[E]thologists have generally ducked the question of what social bonds actually are… It may now be time to engage more directly with the nature of social relationships and the phenomenon of bondedness in animals. We argue that, at least in respect of our understanding of the more intensely social species of animals, progress has been impeded by an over-dependence on the behaviorist stance*—*the claim made by the early twentieth century behaviorists that we can only ever study behavior and not the mind behind the behavior (Dunbar and Shultz, 2010).

A rigid insistence on the observability of a feeling in order for it to gain “ontological status”—that is, for it to be considered a “real thing”—can lead to distortions and possibly deficient conclusions. Consider for example a distinction that some may make regarding *bonding* versus *attachment*. The concept of “attachment” emerged from John Bowlby’s observations of children separated from their parents (Ainsworth and Bowlby, 1991), Harry Harlow’s finding that monkeys would cling to a warmer and cloth-covered wire model of a mother rather than a bare model that also gave milk (Harlow and Zimmermann, 1959), and Mary Ainsworth’s articulation of different “styles” for being attached (Ainsworth and Bowlby, 1991). In the 1970s, increased attention to “bonding” as a discrete concept arose in part due to the hypothesis of a critical period for postpartum women to become *bonded* to their infants and develop healthy maternal care behaviors (Klaus et al., 1972). The net effect of this conceptual evolution has been the idea that “while caregivers may develop bonds with their infants, infants become attached to their caregivers” (Gangestad and Grebe, 2017).

Yet at face value, this distinction is problematic. In any culture, are there healthy mothers for whom bondedness to their child is not a very special and strong feeling? If we use a different term to describe the infant’s experience, though, we undermine recognition of a *reciprocal* special and strong feeling—a feeling which disappears to science to the extent that we focus, instead, on the “behaviors of attachment.” In other words, this labeling may be one “mile marker” in the path of behaviorism to create lacunae in our understanding of social affiliation.

Well before modern science, in colonial North America there were already indications of the erosion of the sense of kinship, recently pointed out by Graeber and Wengrow (2021). The special character of the bonds of indigenous Americans was well recognized. For example, children of colonists were sometimes kidnapped by Natives in situations of border skirmish, yet after being rescued by colonists these white children would in some cases prefer to return to the Native communities. The Frenchman J. Hector St. John de Crevecoeur wrote that “the Indians must possess a ‘social bond singularly captivating, and far superior to anything to be boasted of among us’” (Graeber and Wengrow, 2021). And thus our point here is that if there is to be a genuinely universal affective neuroscience, then it may need to recalibrate its own mentality so that it can admit and better characterize some subtleties of feeling which may have lost a degree of salience on the path that humanity took—which was sometimes fraught—toward the WEIRD mind and the modern world.

### 2.2 *Jeong:* the feeling of bondedness

One blind spot in the universalistic thinking of the WEIRD mind is that it may overlook how all forms of human feeling, thinking, and behavior are nonetheless dependent on local physical conditions. In his classic work, *Guns, Germs, and Steel*, Jared Diamond brought attention to features of the earth as main actors in human history when he contended that Eurasia’s contiguous longitudinal land mass, which eased movement across long distances, was a critical factor in the success of the West (Diamond, 1999). Subsequently, Richard Nisbett has proposed that because mountainous descents to the sea favored herding, hunting, fishing, and trading, Ancient Greece encouraged a culture of commercial engagements which required extensive dialogue, eventually leading to a “folk metaphysics” that valued thinking about “pure abstractions” (Nisbett, 2004). By contrast China consists of fertile plains, low mountains, and easily navigable rivers which made early central control easier, so that the mind focused on practical applications and social relationships. To the early Chinese mind, “thinking for its own sake” led to trouble, and was frowned upon.

Though Korea exists on a peninsula, historical factors arguably guided the nation to develop a more “insular” mentality (Cumings, 2005). In contrast to Italy, Iberia, or Arabia, which became centers for outward expansion and trade, persistent threats from stronger foreign powers may have led Korea toward a more inward orientation. Without air travel, it is impossible on a moment’s notice to move beyond a certain distance, and an effect of these boundaries is to direct attention towards community feelings and bonds. As with islander cultures, Korean emotion seems to entail awareness that everyone is in the same “boat”—even if there are several seating classes.

**Figure 1:**
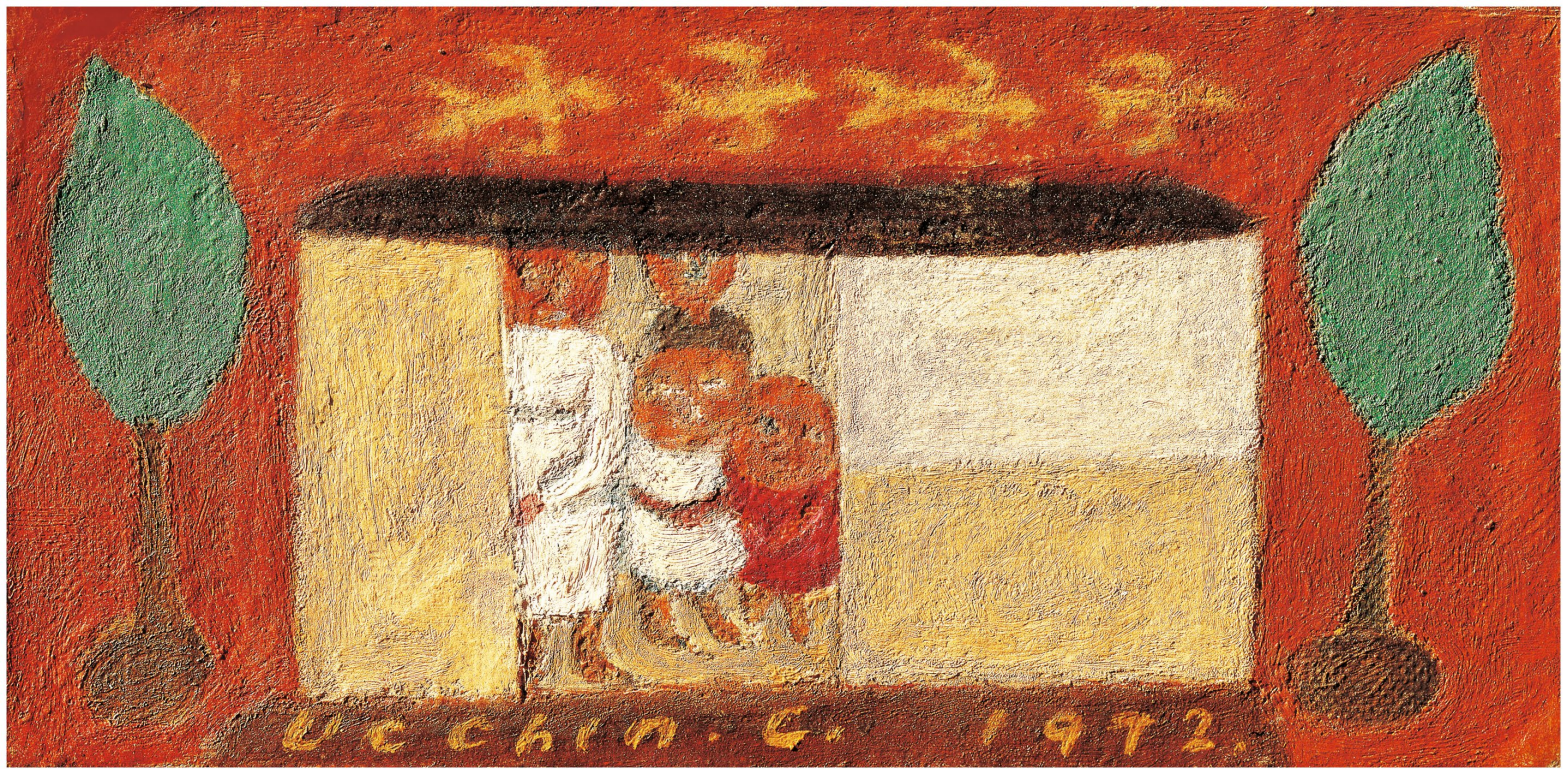
*Family* (1972), by Chang Ucchin (1917–90). The simple, child-like renderings of a nuclear family, their tiny house that apparently is nonetheless big enough, and the protective trees and large birds convey the warmth of *jeong*. Much of Chang’s art reflects traditional Korean *jeong* becoming filtered through selected Western-influenced modernist styles. ©2024 CHANGUCCHIN FOUNDATION. All rights reserved.

Historically, *jeong* has been the binding awareness in Korean life. Irene Kim and colleagues define it as a “special interpersonal bond of trust and closeness” (Kim et al., 2006). While *jeong* overlaps with affection, tenderness, compassion, attachment, and love, one author of this paper (Lee) would describe it as a stable and vaguely pleasing viscerality that does not depend on intimacy, that usually arises after some period of physical proximity. When *jeong* grows, Koreans say *jeong deul uhs seum needa*—“the *jeong* is now permeating,” as if a flavor has saturated a fermenting food. *Jeong* may extend to animals, objects, physical spaces, and even abstract ideas, yet in general Koreans are likely to agree that some time must pass before one feels it. The presence of *jeong* brings a sense of fullness or connectedness (Figure 1), and Koreans usually notice that *jeong* eventually flows for whoever and whatever is nearby, and for the environment itself.^1^

For a popular culture illustration of *jeong*—and *haan*, the feeling of loss which we will explore later in this paper—consider the absurdist 2021 internet streaming drama, *Squid Game*. The plot involved desperate, indebted individuals who played children’s games—in the setting of an island—to win a cash prize, with the penalty of loss being a grotesque death. As a satire of economic systems which exalt struggle for survival, it was only its *jeong* that gave it the status of art. Initially, the players mainly felt *jeong* for their families, especially mothers and children that they were desperate to support. Yet eventually *jeong* grew among the players themselves, in conflict with the zero-sum rules. The main character shows his *jeong* in the show’s opening, through his affection for a stray cat, and throughout the sordid games he tries to resurrect withered *jeong* with his childhood friend or to create it freshly with others. His friend reciprocated just enough *jeong* to address him by the honorific *hyung*, “Elder Brother,” but not enough to quit their duel to the death. The North Korean defector and the alienated daughter of a minister unexpectedly realized the *jeong* that had grown between them. The Pakistani worker probably felt the deepest *jeong* of all, and he was the first to take action to save another player, at risk to his own life. It is a feeling of familiarity, comfort, and safety—and a sense that “everyone is in the game together.”

Importantly, *jeong* is a feeling without moral status in the Western sense. That is to say, although one may feel the warmth of a bond as a “good feeling,” *jeong* is not a “good” as a valuation or directive in the image of, for example, Kant’s categorical imperative. While subtle aspects of *jeong* overlap with Confucian virtue or Buddhist compassion for all sentient beings, *jeong* can disrespect individual preferences, and it can also be a tool for manipulation. In its heart and at its best, *jeong* is a kinship bond that can connect all mammals great or small. At its worst, *jeong* leads to tribalism, nepotism, cronyism, and crime families. Hazing rituals build *jeong* in a way that is limited to privileged insiders, and in *Squid Game*, the gangster character maintains *jeong* in his clique through bullying, sexist humor and other forms of degraded “kinship” and control.

To describe the feeling of connectedness—which exists in all cultures and in other mammalian species, and also between many species that exist in familial symbiosis (e.g., humans and their pets)—we propose that *jeong* can be a useful addition to the global lexicon of science and culture. *Jeong* can be a way to denote the felt sense that not only connects one human to another or to their community, it can refer to one’s bondedness to an animal companion, a teddy bear or an old sweater, the physical landmarks of their hometown, or even an office cubicle. Drivers may have *jeong* for their cars, and sailors and pilots may develop *jeong* for their ships and planes. Intellectuals may have *jeong* for certain ideas, and experimental scientists may have *jeong* for their labs. Artists may have a special sensitivity to the *jeong* of aesthetic experience, and later in this paper we will explore some implications of brain opioids for *jeong* and artistic practice.

Though Jean-Marie Gustave Le Clézio, a French Nobel laureate for literature, has stated that the feeling of *jeong* is “untranslatable into French” (Doo, 2016), we contend that the feeling of bondedness is, axiomatically, palpable in any human and likely all mammals, and like all natural feelings it must have a neurobiological foundation.

### 2.3 *Jeong* may depend on endogenous brain opioids

Social behavior relies upon the simultaneous integration of sensory information and the engagement of various reward, decision-making, memory, and motor centers which collectively constitute a “social brain” network. These integrated regions communicate via neurotransmitters and neuromodulators which regulate diverse neuronal populations and modify cellular and regional response patterns to specific social stimuli and thereby influence thought and behavioral output (Chen and Hong, 2018; Dunbar, 1998; Newman, 1999). The systems work in tandem to underpin initiation, maintenance and cessation of social behaviors that can be expressed under an endless variety of environmental contexts.

Importantly, no one molecule is responsible for the entirety of social behavioral expression. Oxytocin, for example, which is released during childbirth, lactation, and sexual completion, has been well recognized for its influence on attachment behaviors in parents and children, and romantic partners (Jurek and Neumann, 2018; Bartz et al., 2011). Serotonin, dopamine and vasopressin are also well studied for their roles in the guidance or management of social affiliation, invigoration of expressions, and regulation of aggressive interactions, respectively (Damsma et al., 1992; Gingrich et al., 2000; Meisel et al., 1996; Donahue et al., 2015; Couppis and Kennedy, 2008; Manduca et al., 2016b).

In this paper we focus on the role of the endogenous opioid system. While this system has been most commonly recognized for its role in antinociception and addiction, it also plays a pivotal role for maintenance of affiliative, cooperative, and other social behaviors (Stein et al., 2007; Le Merrer et al., 2009; Buchel et al., 2018; Mechling et al., 2016; Matthes et al., 1996; Machin and Dunbar, 2011).

Indirect knowledge of the opioid system, and its involvement in pain modulation, reward, and motivation has been around for centuries, however it wasn’t until 1973 that researchers discovered a class of cellular receptors specific for morphine, the pain-relieving and pleasure-boosting compound found in the sticky, gum-like latex of the opium poppy (Brownstein, 1993). Opioid binding sites in the central and peripheral nervous system were identified and later described as a family of trimeric g-protein coupled receptors including mu, kappa, and delta (Brownstein, 1993; Al-Hasani and Bruchas, 2011; Bodnar, 2017). Later, the main endogenous ligands Met- and Leu-enkephalin were identified, with the discovery of dynorphins and endorphins soon to follow. Together these brain opioids act as neuromodulators that carry messages from one neuron to another, changing the gain on neuronal signaling, and as hormones that signal widely through the bloodstream.

Upon activation by their endogenous peptides or by exogenous opiate drugs, the Go/Gi coupled opioid receptors modulate intracellular effectors and pathways leading to short term inhibition of neuronal activity and a decrease in neurotransmitter release (Al-Hasani and Bruchas, 2011). Opioid receptors and their peptide ligands, the endorphins, enkephalins and dynorphins, form an extensive heterogeneous network throughout the central and peripheral nervous system that are most notable for their involvement in pain and reward processing (Le Merrer et al., 2009). Endorphins predominantly bind to and activate the mu opioid receptor (MOR) and as such they are linked to the consumatory reward system, eliciting feelings of pleasure, liking, and gratification (Le Merrer et al., 2009; Buchel et al., 2018; Gosnell et al., 1986; Ragnauth et al., 2000; Berridge et al., 2009; Filliol et al., 2000; Richard and Fields, 2016; Darcq and Kieffer, 2018; Lutz and Kieffer, 2013; Castro and Bruchas, 2019). For example, MOR stimulation in specific regions in the nucleus accumbens (NAc) and the ventral pallidum known as “hedonic hotspots” amplify the hedonic impact or “sensory pleasure” of palatable food (Castro and Berridge, 2014).

While “consumption” as it relates to social behavior is physically less structured than feeding, there are clear ethological delineations in the behavioral sequences leading up to, during the maintenance of, and extending beyond the cessation of reciprocated and non-reciprocated social contact. Early behavioral studies that have inadvertently investigated MOR-mediated hedonic modulation of social interaction have found that stimulation of MORs specifically in the NAc enhances social play between juvenile rats (Trezza et al., 2010) and primates (Guard et al., 2002), though extensive mapping of hedonic hotspot regulation of adult social interactive behaviors has not been conducted. Nonetheless, opioid activation within these regions may enhance the enjoyment of social contact, potentially driving the development of *jeong* or bondedness or kinship, and in the following paragraphs we summarize some of the key findings that are supportive of this hypothesis.

Soon after their discovery in the early 1970’s, endorphins were proposed as the neurochemical mechanism motivating romantic and parental behavior in humans based upon intuitive and observable similarities between opioid drug addiction and romantic relationships (Machin and Dunbar, 2011; Panksepp et al., 1980). Studies of opioid modulation of social behavior began with Jaak Panksepp, who developed the brain opioid theory of social attachment, or the BOTSA. This theory posits that mu opioid receptors underlie the hedonic aspects of social connection (Panksepp et al., 1980), and it was inspired by a set of key observations. In humans, social affiliation elicits feelings of pleasure and of social connection which Panksepp related to increased opioid tone, based on the behavioral and emotional characteristics similarly exhibited by those addicted to opiate drugs. Additionally, Panksepp related the profound distress following social separation or isolation to a decrease in opioid tone, given the behavioral similarities to drug withdrawal, with both being promptly alleviated by restoration of social contact or administration of the drug of abuse (Panksepp et al., 1980).

Subsequent basic research has provided evidence in support of the BOTSA. Foundational studies in animals have highlighted a key role for MORs in socio-sexual behavior, social attachment—specifically in maternal attachment behavior and pup separation distress—and juvenile play behaviors (Guard et al., 2002; Normansell and Panksepp, 1990; Manduca et al., 2016a; Vanderschuren et al., 1995; Achterberg et al., 2019; Inagaki et al., 2016). Systemic morphine administration selectively enhances social play behavior in juvenile marmosets, whereas naloxone, a MOR antagonist, subtly decreases this behavior (Guard et al., 2002; Achterberg et al., 2019; Vanderschuren et al., 2016). Additionally, various MOR agonists and antagonists administered systemically have repeatedly been shown to increase and decrease social play in juvenile rats.

In humans, dysregulation of MOR signaling is reported in a variety of neuropsychiatric conditions including autism spectrum disorder (Pellissier et al., 2018), major depression (Kennedy et al., 2006), anxiety (Nummenmaa et al., 2020), borderline personality (Prossin et al., 2010), and schizophrenia (Ashok et al., 2019), all of which present with degrees of social behavioral perturbation. Interestingly, healthy adult humans with the A118G variant of the mu opioid receptor gene (*Oprm1*) display greater sensitivity to social rejection (Way et al., 2009) as well as greater social hedonic capacity (Troisi et al., 2011), and studies that have endeavored to manipulate MORs in humans—either by blocking or activating the receptor with exogenous drugs—have found a large impact on social affiliation. For example, among women with high levels of social affiliation, those who took naltrexone, a MOR antagonist, showed lower feelings of warmth and social connection than peers who took placebo (Inagaki et al., 2019), and naltrexone administration also reduced feelings of social connection in men and women who read loving messages from friends and family (Inagaki et al., 2016).

Critically, several of these studies have highlighted the role of MORs for the *rewarding* aspects of social interaction, that is to say for increasing the time spent maintaining or engaging in play or affiliative behavior, rather than for the motivational aspects. For example, MOR activity seemingly does not affect the rate at which an animal acquires a spatial learning task rewarded with play, and by contrast morphine or naloxone treatment leads to longer or shorter duration of play, respectively (Normansell and Panksepp, 1990). In a monogenic model of autism (Oddi et al., 2013), mice lacking the *Oprm1* gene that encodes the MOR (*Oprm1*^−/−^) display reduced maternal attachment in mouse pups (Moles et al., 2004), blunted social reward in juveniles (Cinque et al., 2012), impaired abilities to remember familiar social partners in a social memory test, and profound deficits in interactions with social partners (Becker et al., 2014), and heterozygous MOR knockout mice (*Oprm1*^+/−^) display similarly profound social deficits (Toddes et al., 2021). During freely moving social assays, both *Oprm1*^−/−^ and *Oprm1*^+/−^ mice not only spend less time socially engaged overall, but also exhibit significantly perturbed social behavioral repertoires including fewer reciprocal social interactions with a wildtype partner, though not exploratory ones (Toddes et al., 2021). These findings demonstrate that MOR knockout mice are not necessarily impaired in initiating social interactions, suggesting that the commencement of social affiliation may depend on neural processes that are independent of opioid signaling, and this temporal sequence could explain why *jeong* does not develop, traditionally, until later in the process of forming a state of kinship.

Furthermore and even more intriguingly, the differences in social behavioral responses by the mutant animals are clearly detectable by wildtype mice, who alter their own social behavior when faced with the atypical social partner. When wildtype mice are given the choice in a real-time social preference assay between spending time in a chamber containing an *Oprm1*^−/−^ mouse or a chamber containing another wildtype, the freely moving wildtype mouse spends significantly less time with a knockout mouse over another wildtype animal. Parallelwise, *Oprm1*^−/−^ mice preferred the social chamber containing a novel *Oprm1*^−/−^ over a wildtype mouse. Taken together, these studies may provide some *prima facie* neurobiological validation for the idea that nuances in affiliative social preference or attachment behaviors are not only trans-cultural they are trans-species (Shah et al., 2013; Beery et al., 2021); they do not require an advanced neocortex; and they may be at least partially influenced by genetic differences in the mu opioid receptor—though regarding this latter point we are quick to emphasize that there are environmental influences on all forms of gene expression, and we raise this topic again in Section 3.4. At all events these data suggest that there may be opioidergic correlates for different degrees of sensitivity to *jeong* [which might also correlate with anxious versus avoidant (Nummenmaa et al., 2015) attachment patterns]; or for the everyday observation that individuals often self-aggregate by preference for bonding styles.

Unsurprisingly, human imaging studies using positron emission tomography (PET) have found extensive MOR activation under conditions of social acceptance. During PET scanning, social acceptance was associated with MOR activation in the amygdala, anterior insula, and left ventral striatum, and with MOR deactivation in the midline thalamus and subgenual anterior cingulate cortex (Hsu et al., 2013; Hsu et al., 2015). A greater degree of MOR activation in the anterior insula and ventral striatum, which are regions linked to mood, empathy, and reward, predicted the desire to interact socially and individuals reported feeling more “happy and accepted.” Notably, it was also found that research participants who were in pre-existing relationships had greater MOR activation during social acceptance, suggesting that being in a social pair bond may facilitate MOR responsiveness to others, amplify social enjoyment, and promote feelings of security in the presence of others.

Yet perhaps also unsurprisingly, MORs are also activated during social rejection and in ways that are consistent with their antinociceptive functions throughout the brain. While there are some areas of overlap in MOR activation during states of acceptance versus rejection, there are several key differences. During social rejection, MOR activation occurs specifically within the ventral striatum, amygdala, midline thalamus, subgenual anterior cingulate cortex and periaqueductal gray. These regions are canonically associated with stress, threat detection, fear and the alleviation of pain, correlating with feeling “sad and disconnected” (Hsu et al., 2013; Hsu et al., 2015). These functional results align with MOR antagonism experiments where individuals given naltrexone increasingly felt disconnected from others compared to placebo (Inagaki et al., 2016), and interestingly this pattern is highly similar to MOR activation shown in acute physical pain (Hsu et al., 2015). This finding supports a growing body of literature on the overlapping neural pathways in pain and social processing and the involvement of the endogenous opioid system in the modulation of emotional pain.

From an evolutionary perspective, the dissolution of social bonds and the resulting social isolation could have endangered our predecessors no less than a broken bone. Experiencing pain at the dissolution of social bonds could have motivated the individual through negative reinforcement to re-establish social connections, thus reinstating safety through numbers. This concept of “social pain” was first posited by Herman and Panksepp, who argued that “brain circuits for separation distress represent an evolutionary elaboration of an endorphin-based pain network” (Herman and Panksepp, 1978). Following separation from their mothers, administration of morphine to chick or mouse pups led to alleviation of distress vocalizations, indicating a potential decrease in the animal’s experience of pain upon social separation. Further, it is well documented that social isolation enhances the perception of physical pain, while positive social experiences—which will contribute to the feeling of connectedness or *jeong*—may act as a “buffer” against it (Martin et al., 2014), and these data collectively emphasize the overlapping role of the opioid system in pain and sociality.

In other words, states of social affiliation, bondedness, or *jeong*, may lead to opioid receptor activation in limbic regions associated with reward and deactivation in regions associated with threat and pain detection, creating a net effect of attenuated perception of physical pain and a subjectively enhanced sense of safety. Likewise, social rejection, disconnectedness, or what we will later introduce as *haan,* may subtly diminish opioid activity in reward related limbic regions and activate opioidergic processes in regions associated with pain and stress (Massaccesi et al., 2022), exacerbating the perception of physical pain. We will later explore how attempts to re-establish social connection during these periods of shifting or diminished opioidergic dynamics may contribute to maladaptive social behavioral patterns.

To reiterate, no single neural system controls the whole of sociality, nonetheless we endorse the assessment given in A. J. Machin and R. I. M. Dunbar’s excellent review of the BOTSA: “while oxytocin, vasopressin, dopamine and serotonin may be implicated in their onset, the endogenous opioids may play the maintenance role which is vital for, amongst other things, stable long-term relationships and the rearing of psychologically healthy, socially adept human beings.” (Machin and Dunbar, 2011) MOR hedonic hotspots, specifically in the NAc, may work in tandem with oxytocin and serotonin receptor systems to modulate the “hedonic gain” of social interactions contributing to the development of social preferences and maintenance of social bonds. MOR activation in humans positively predicts the desire to interact socially (Hsu et al., 2013; Hsu et al., 2015), and these findings may further reinforce that endogenous opioid activity contributes, even indirectly, to a variety of social engagement and modulatory effects.

**Figure 2:**
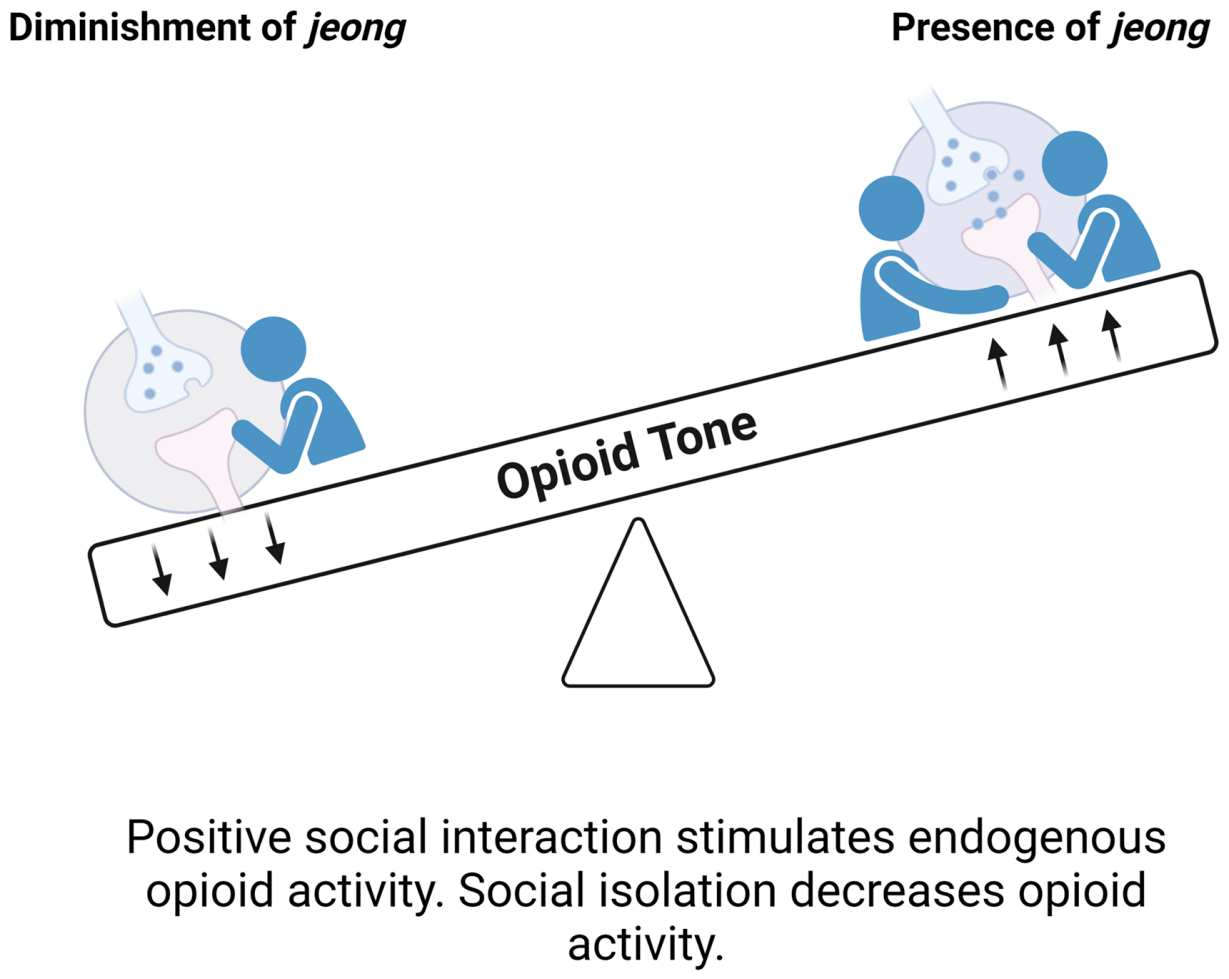
According to the brain opioid theory of social attachment (BOTSA), feelings associated with bondedness or social attachment depend on endogenous opioids, especially endorphins and enkephalins (right side of figure). Increased tonic signaling from endogenous opioid molecules may be responsible for the subtly positive feeling of the presence of *jeong*.

Taken together, phenomenology and empirical findings lead us to conjecture that endogenous opioids play a sustaining and constitutive role for the ultra-subtle, warm and tonic awareness of connectedness that is *jeong* (Figure 2). To further explore and test the ideas in this section and throughout this paper, more research evaluating *in-vivo* opioid dynamics during the initiation, consumption, and cessation of social interactions will be necessary. Additionally, studies exploring the mechanistic interactions between various neurotransmitters and modulators (e.g., oxytocin, dopamine, and others) with the opioid system in the context of typical or atypical interactions, the development of various social behavioral patterns, and the vulnerabilities introduced by social isolation or rejection could also greatly advance our understanding of the underpinnings of human sociality.

Finally, the notion of *jeong* extends the reach of the BOTSA by recognizing how the sense of bondedness is not limited to human attachments, given that *jeong* may be felt for non-human animals, inanimate objects, physical spaces, or even abstract concepts.^2^ If we accept common usages of *jeong* awareness, we can generalize the biochemical processes inherent to opioidergic modulation of social affiliation—increased mu opioid receptor binding leads to increased feelings of connectedness and cooperation—toward a general neurobiological model for a feeling of warmth and kinship that can extend to any living being or non-living entity. Research on neuromodulatory systems that encode social salience may also benefit from studying how social information transfer and bondedness can occur through artistic experience, as we speculate later in this paper. Yet to further elucidate the mechanisms of *jeong*, we must first consider what occurs when it is missing, or when affiliative bonds to people, objects or places have dissolved.

## 3 A new model for emotional loss

### 3.1 Original *haan*—a subtle feeling of missingness

Though the BOTSA was first formulated as a way to explain the positive feeling of social bonds including the pleasure of romantic relationships; and while we have leveraged it in this paper to explain the feeling of *jeong*; and while we leverage it later to explain some clinical conditions and public health findings; it also points us, here, to consider another and entirely different feeling. Specifically, consider that natural withdrawal from a *subtle* level of endogenous brain opioid release may entail *its own subjective experience*. If the ending of an intense romance can create a negative and aversive feeling that is not unlike the dysphoria of sudden cessation of morphine, then *jeong* diminishment ought to bring its own natural feeling, one that creates its own awareness of missingness or emptiness, and again there is some evidence for differential patterns of mu opioid receptor activation that associate with social acceptance versus rejection (Hsu et al., 2013). Yet because *jeong* withdrawal is not so intense as an event of outright social rejection, or what one feels if a lover has left, this “closer-to-zero” state of emotionality—when felt at a barely perceptible level—ought to have its own phenomenological character, if an individual is on the “lookout” for it. We contend that it does, and we call it *original haan* so as to differentiate it from a form of *haan* to be discussed in the next section. We postulate—on both theoretical grounds and introspection—that original *haan* is an overlooked yet critically valuable awareness, because it serves as a kind of “reset to zero” for the sense of connectedness itself.

We conceptualize original *haan* as a natural feeling palpable to any neural system that depends on endogenous opioids for the stable sense of connectedness. A dog that misses its human, for example, is capable of feeling original *haan*. It is a recognition of space itself, and it precedes cognitive valuation or the creation of narrative that may seek to explain the feeling of missingness. Perception of original *haan* may be helped by “no-thinking” traditions such as zen, which have a literary equivalent in the insight of John Milton—that “The mind is its own place, and in itself Can make a Heaven of Hell, a Hell of Heaven” (Milton, 1667). And thus original *haan* also encourages creativity through the “beginner’s mind” or a point of freedom from preconceptions.

Our description of original *haan* overlaps with an awareness that has been compared, by some Korean psychologists, to the cyclical, evolutionary engagement of object-background relationships that is fundamental to Gestalt therapy. That is to say:

When we perceive an object, we can experience that the part we are interested in becomes the center of perception, and the rest recedes into the background… the phrase ‘forming a Gestalt’ means ‘an individual perceives the most important desire or emotion at a certain moment and brings it to the foreground.’ Healthy individuals can clearly and strongly form Gestalts that are important to them at every moment and bring them to the foreground… When an object fails to form a Gestalt, or when it does form a Gestalt but its resolution is prevented… [then there can be] an ‘unresolved Gestalt’ or ‘unfinished business’… thus hindering the adaptation of the individual… Since unresolved issues always try to come to the foreground, they always appear ‘here and now’, and therefore the individual only has to notice them rather than avoid them… In Korean terms, it has the same meaning as *haan*. (Room/International Institute of Psychology, n.d.)

Just as the invention of zero enabled advances for mathematics, we propose that original *haan*—an experience of a trace reduction of opioidergic tone that is too small to be grossly dysphoric, that stems from any perception of “unfinished business” in a relationship, work product, artistic experience, or any other endeavor—can help to define a state of “neutral emotional missingness or emptiness” and that it may be a help to future progress in affective neuroscience.

With the above said, most *Homo sapiens* are not zen practitioners, and most of us are quick to apply *thinking*—to include conscious valuation and explanatory causal narrative—toward any withdrawal of even a semblance of pleasure. Original *haan* or the barely perceptible awareness of missingness—which could be due to a miniscule change in receptor binding for someone who, for whatever reason, is sensitive to it—may transform into its own distinct affective state. This feeling, which is also called *haan*, has attributes which merit its recognition as a different kind.

### 3.2 The transformation of original into constructed *haan*

Another definition for *haan* comes from an outsider’s perspective, in the tradition of Alexis de Tocqueville or other foreign observers who are sometimes adroit interpreters of a different culture. While enjoying a visit to a street food market, the celebrity chef Anthony Bourdain asked his guide, “How come all the Korean guys are so tormented? They’re all carrying around some unseen weight” (Anthony Bourdain on Han: parts unknown (S. Korea), 2018). After hearing how *haan* stems from a sense of injustice at the historical wrongs Koreans have suffered, Bourdain described the feeling as “a mixture of endurance, yearning, sorrow, regret, bitterness, spite, hatred, and a grim determination to bide your time until revenge can at last be exacted.” To differentiate this intense and regressive set of feelings from the subtlety of original *haan*, we propose the concept of *constructed haan.*

Consider again the *Squid Game* characters. They did not wear their *jeong* on their sleeves. Instead an exploited worker was angry at an evil boss; a defector hated a trafficker who had double-crossed her; a mid-life investment banker regretted his financial losses; and a former labor organizer was bitter from being separated from his daughter and his absent job prospects. An old man with a brain tumor felt a hollowness which allowed him to resonate with the others, even if he was less possessed by poisonous intentions. These feelings - resentment, grievance, barely suppressed anger - mix into variations on what we call the constructed kind of *haan*.

The critical difference between the two kinds of *haan* depends, again, on the human power of valuation. Original *haan* is a natural feeling, not so different from thirst or itch. As to constructed *haan*, a simple and mammalian feeling of loss turns into resentment or grievance when *Homo sapiens* apply cognitions, narratives, and especially moral appraisals. Human thinking can create belief systems around any circumstance. Through thinking, we can define behaviors as bad or evil, and identify a particular person or event as the cause of whatever—or whomever—we lost. The moment we make that assignment is when original *haan* transforms into a different kind of feeling, or what we label as constructed *haan*. It is through rumination around what seems missing, that we generate resentment, grievance, anger, or related emotions which on their own or in mixtures have potential to turn toxic. Accordingly, mindfulness meditation or other self-care strategies that support the *witnessing* or neutral awareness of the contents of consciousness including thoughts or feelings, rather than egoic “identification” with them, may be a way to benefit from the recurrence of original *haan* and to avoid its constructed cousin.

At this juncture, we emphasize that this paper presents novel conceptions of *jeong*, original *haan*, and constructed *haan*, with the goal of expanding the denotations, connotations, and applications of feeling-concepts which may be of global value and beyond what some Koreans themselves may recognize. In other words, we question the view that *haan*—whether original or constructed—is mainly a consequence of the colonial era (Kang, 2022), and we even dare to challenge the attestation of the novelist Han Kang, Korea’s first Nobel laureate for literature, who purportedly once stated to a fellow writer that “[*Haan* and *jeong*] are not my topics. I’m past that generation.” (Doo, 2016) Our conceptualization of these feelings, drawn from both phenomenology and neurobiology, is such that they are not only thematic to modern and variegated Korean cultural expressions, albeit often in ways that may be extremely subtle; they are part and parcel of life as a human being.^3^

### 3.3 The brain opioid and vasopressin theory of original and constructed *haan* (BOVTOCH)

In this section we present speculative hypotheses that derive from disparate fields of research. There is a need for more preclinical and human studies that directly explore the interactions of endogenous opioids with other neuropeptide and neurotransmitter systems. Nonetheless we consider that there is sufficient data to draw inferences that could help to explain some highly prevalent patterns of social vulnerabilities along with both adaptive and maladaptive responses.

If the BOTSA can help us understand how original *haan* emerges in the wake of loss, it says nothing about how original *haan*—a subtle awareness of missingness—may transform into an entirely different kind of feeling. For a fuller biological model to explain how a sense of emptiness has potential to transform—through thinking, cognitive appraisals, and moral assignments—into constructed *haan* that has the potential to fuel aggressive actions, we may require another process or factor. The hormone vasopressin may be a critical contributor.

While the genetic structure of vasopressin is very similar to oxytocin, a molecule traditionally considered as pro-affiliative toward in-group members, the role of vasopressin is more opaque. Both vasopressin and oxytocin derive from the even more primitive molecule vasotocin, which is found in reptiles and can be measured in the mammalian fetus (Carter, 2017). As genetic descendants of vasotocin, vasopressin came first, perhaps two hundred million years ago, and oxytocin was later by perhaps another one hundred million years. Arginine vasopressin, also known as antidiuretic hormone, is synthesized in the magnocellular cells of the hypothalamic supraoptic nucleus (SON) and the paraventricular nuclei (PVN). Release of vasopressin into the bloodstream from the hypothalamus causes water retention, increasing blood pressure. Additionally, vasopressin released from the PVN increases the production of adrenal stress hormones by stimulating the hypothalamic–pituitary–adrenal axis (HPA) and thereby modulating stress responses. Taken together, these functions are consistent with the idea that vasopressin may have emerged as a molecule that supported basic physical survival in the most ancient species of animals (Carter, 2017).

Vasopressin is produced in populations of magnocellular neurons located in the bed nucleus of the stria terminalis (BNST), medial amygdala (MeA) and suprachiasmatic nucleus (SCN) whose extensive projections have been shown to regulate essential functions including aggression, affiliation, memory, anxiety, and depression. The projections from these regions also extend throughout the social brain network, positioning vasopressin as a key regulator of various social behaviors (Albers, 2012; Phelps et al., 2017). Intriguingly and importantly for a new theory that we present later in this section, a number of studies have found that endogenous opioid peptides inhibit vasopressin-containing neurons both within the hypothalamus and within limbic regions of the brain (Zhao et al., 1988; Russell et al., 1992). By contrast the short form opioid peptide dynorphin, which shows binding affinity to both mu and kappa opioid receptors, is co-expressed in vasopressin magnocellular neurons (Pfaff and Joels, 2016; Lightman and Young 3rd, 1987; Lightman and Young, 1988), and the presence of an opioid agonist in vasopressin neurons indicates that vasopressin receptor activation in limbic regions may co-occur with subtle activation of opioid receptors, such as the kappa type, that are linked to dysphoria and depressed mood (Kieffer and Gavériaux-Ruff, 2002).

Notably, vasopressin has been shown to be a sexually dimorphic molecule both functionally and anatomically, such that vasopressin’s role in aggressive social interactions, social stress, and anxiety can have opposing functions in males and females. Canonically, vasopressin activation has been shown to invigorate aggressive interactions in male animals whereas vasopressin blockade inhibits aggressive interactions in females (Rigney et al., 2023). In men, intranasal administration of vasopressin reduces the perception of friendliness in the faces of unfamiliar men (Thompson et al., 2006), promotes aggressive behavioral responses in an economic game (Kawada et al., 2019), promotes autonomic responsiveness to social threat and anxiety (Born et al., 2002), and increases the experience of social stress (Ebstein et al., 2009). Additionally, high cerebrospinal fluid vasopressin concentrations are positively correlated with a life history of aggression in male subjects (Coccaro et al., 1998), and high blood plasma vasopressin concentrations are highly correlative to male patients with depressive disorders (van Londen et al., 1997).

Thus while its effects are many and varied, vasopressin is a likely candidate for the potential underlying mechanism contributing to the development of constructed *haan* in men, where social stressors may lead to the activation of both the hypothalamic and limbic vasopressin system, causing an increased perception of social threat in others and thus the invigoration of aggressive social behaviors. In male preclinical models of pathological aggression, it has been shown that positive fighting experience, or winning aggressive interactions, provides permanent reward to the winners leading to repeated acts of aggression (Smagin et al., 2022). Interestingly, male mice who develop pathological aggression display significant upregulation in the gene encoding for kappa opioid receptors, indicating alterations in reward processing and an entrance into dysphoric states (Smagin et al., 2022; Golden et al., 2019). The dynorphin peptide, which binds kappa opioid receptors, is co-released with vasopressin (Brown et al., 2007) indicating a potential crossover of these two systems following the transition into a maladaptive aggressive state which could be a driver of constructed *haan* in males.

We hypothesize that in males, under normal conditions, robust levels of endogenous enkephalins and endorphins—when we feel enough *jeong* from positive social interactions—may prevent the release of aggression-related vasopressin. Conversely, if endogenous enkephalin and endorphin levels fall—if *jeong* becomes weak or disappears due to negative interactions, or social rejections—then vasopressin may rise, promoting kappa opioid binding, dysphoria, and an engagement in agonistic behaviors that aim to recover the lost *jeong*. Permanent winners of repeated aggressive acts show dysregulation in the natural reward system, impairments in social communication and sociability and persistence of behavioral changes even after deprivation of agonistic interactions (Golden et al., 2019; Golden et al., 2017; Golden and Shaham, 2018; Aubry et al., 2022). This data may indicate why constructed or toxic *haan*, once developed in men, may be a hard cycle to break (Yan et al., 2024).

**Figure 3:**
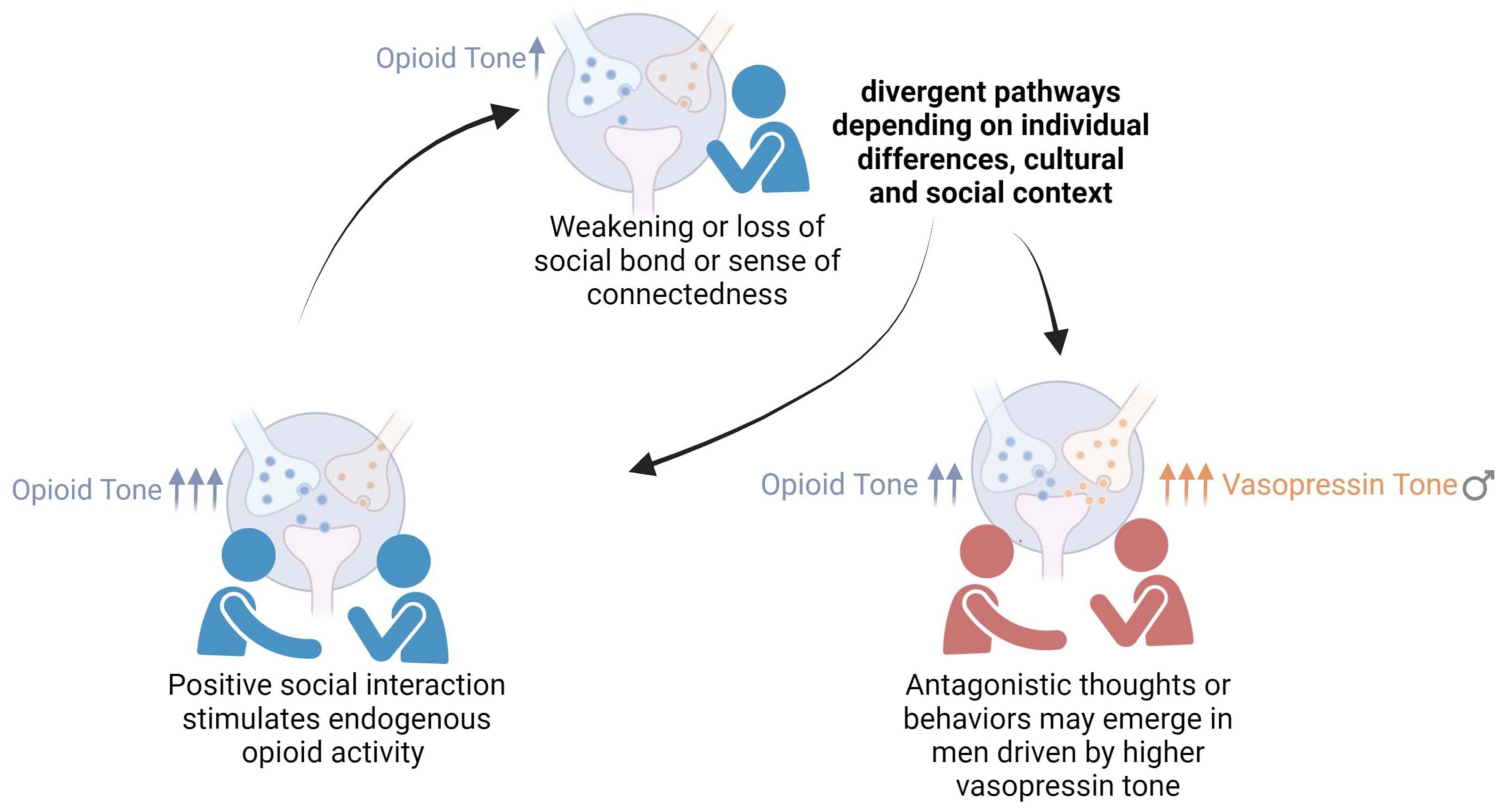
The brain opioid and vasopressin theory of original and constructed *haan* (BOVTOCH). A subtle reduction in the feeling of *jeong* (bottom left) is, potentially, mediated by a decrement in endorphins and enkephalins that leads to a subtle state of “missingness,” or original *haan* (top center), that has neutral status from a human valuative or moral perspective. Through cognitive appraisals (dependent on cultural or individual belief systems and narratives), original *haan* can become constructed into grievance, resentment, suppressed anger, endurance or related feelings which usually, though not always, have a hostile tenor (it can also lead to “pure” sorrow or sadness). The transition from original to constructed (and sometimes toxic, bottom right) *haan* may be promoted by vasopressin, a “fight or flight” neuropeptide which increases blood pressure and conserves water. In animal studies vasopressin is shown to be inhibited by endorphins and to foster aggression in males. Evolutionarily, its social role may be to motivate recovery of a bond, felt as *jeong*.

Taken together, these mechanisms suggest a companion to the BOTSA. The brain opioid and vasopressin theory of original and constructed *haan*, or the BOVTOCH, proposes that original *haan*—a feeling of pure emptiness or loss—arises from a reduction of tonic brain opioid activity (Figure 3). Less endorphin and enkephalin release could disinhibit magnocellular neurons, permitting an increase in vasopressin. If vasopressin surpasses a threshold in males—alongside, potentially, cognitive appraisal patterns that assign blame for how the state of missingness came to be—then original *haan* may transform into its constructed and in some cases toxic counterpart.

In females, vasopressin may still be involved in the progression of original into constructed *haan*, yet the mechanisms guiding this behavioral transition could be different. Unfortunately, there is a dearth of research regarding the role of vasopressin in female aggression, stress, and anxiety due to the historic exclusion of females from basic medical research. Studies that have been conducted evaluating female non-maternal aggression have uncovered sex specific differences in the key neural circuitry controlling offensive aggression (Newman et al., 2019; Terranova et al., 2017). As opposed to male animals, *activation* of vasopressin receptors in the hypothalamus of female animal models reduces aggression toward females, while *blockade* of vasopressin receptors increases inter-female aggression (Oliveira Ve De et al., 2021; Oliveira Ve De et al., 2019; Borland et al., 2020; Terranova et al., 2016). Data is unavailable regarding vasopressin activity in female aggression toward males, to our knowledge. At all events, in the next section (3.4) we clarify why studies of animal aggression need careful handling before extrapolation to humans.

Interestingly, newer research has found that the sex differences in vasopressin’s influence on social behaviors can also be observed in humans. Vasopressin intranasal administration enhances female perception of friendliness of unfamiliar women, stimulates affiliative facial motor patterns and improves cooperation behavior (Feng et al., 2015); by contrast, a Japanese study (Kawada et al., 2019) showed that inhalation of vasopressin led to more preemptive strikes by both males and females during an economic game—whose design, importantly, entailed no face-to-face contact; and a Chinese study (Feng et al., 2020) has demonstrated that vasopressin administration escalates dishonesty in women but only if the dishonesty is altruistic in nature (i.e., benefiting another person). These studies further demonstrate that the effects of vasopressin on male and female social behavioral regulation are likely context as well as sex dependent. While more research evaluating the neurochemical control of female aggressive interactions is needed, current studies do indicate essential roles of vasopressin in the regulation of female aggression, if through different mechanisms.

The complex role of vasopressin in social behavior is affected by social experience, motivation, and hormonal background, therefore its effects may depend entirely on a highly unique and changing milieu that encompasses both the internal and external condition of any given individual or group. This context dependency may help explain why some may be more susceptible to developing constructed *haan* following social stressors, whereas others may display more resiliency or constructiveness in how they transform their original *haan* (e.g., creatively taking care of “unfinished business” as might be encouraged by Gestalt approaches). Given the complexity of the human brain and its capacity for social organization, there are countless ways to increase enkephalin and endorphin levels—to create *jeong* that could prevent the release of vasopressin and thereby help to inhibit aggression. And there are also countless ways that humans may cause endogenous opioids to fall—so that *jeong* weakens or disappears, and vasopressin rises to invigorate behaviors that aim to recover the lost *jeong*.

### 3.4 Borderline personality disorder: a condition of *jeong* and recurrent *haan*

In this section we leverage the BOTSA and BOVTOCH to postulate that neuropeptide dynamics are critical drivers of pathological behavior in the context of borderline personality disorder (BPD). A diagnosis that carries sharp stigma, BPD is defined by a complex profile (American Psychiatric Association, 2013) that includes some combination of hypersensitivity to rejection, impaired sense of self, intense fear of abandonment and frantic or aggressive attempts to avoid it, impulsivity and risk-taking, chronic sense of emptiness, inappropriate displays of anger, propensity to self-injury or suicidality, rapid and extreme swings in the valuation of others, unstable moods, and a general pattern of instability and chaos in relationships. BPD prevalence has been estimated at roughly 2, 6, 11, and 22 percent of general community, primary care, psychiatric outpatient, and psychiatric inpatient populations, respectively (Leichsenring et al., 2023). While many potential neurobiological causes have been explored (Gunderson et al., 2018; Perez-Rodriguez et al., 2018), studies generally show transdiagnostic findings (e.g., in imaging studies, differences in fronto-limbic and default mode networks (Schmahl and Bremner, 2006) that are also seen in other psychiatric disorders).

Following Stanley and Siever (2010), we theorize that the core interpersonal (and intrapersonal) dysfunction in BPD relates to endogenous opioid activity deficit or hypersensitivity, and this model has received some validation from studies of patients with BPD which show differences in pain threshold, higher use of opioid medications, and differences in opioid receptor binding demonstrable through neuroimaging that could represent a compensatory response (Perez-Rodriguez et al., 2018). The mammalian need for *jeong* mediated by endorphins and enkephalins (BOTSA) may, in some vulnerable individuals, become amplified so as to cross a threshold of acceptability of some given societal norm. When such an individual experiences a loss of their sense of bondedness, or even perceives a threat to it, then *haan* mediated by a decrement of opioids and alterations in vasopressin availability (BOVTOCH) may motivate behaviors that have features of aggression.

The example of BPD provides an opportunity to highlight an advantage of the language of *jeong* and *haan* when integrated with the BOTSA and BOVTOCH: for the most part, the concepts do not necessarily imply “abnormality.” It is possible to characterize behaviors such as sensitivity to rejection, fear or anger related to abandonment, willingness to act and take risks, and readiness to see “both sides” of others as natural expressions of mammalian instincts to preserve a bond. The *jeong* and *haan* of the BOTSA and BOVTOCH encourage us to conceptualize bonding-related neuropeptide dynamics as necessary to both survival and thriving. This alternative schema might help us to more accurately—and proactively—observe and engage some relational behaviors without fear of applying stigma-laden labels, both increasing our capacity to “call them out” when they do cross a line, and possibly helping to clarify how and in what way, precisely, one defines “healthy bonds and boundaries,” and who participates in the making of those definitions.

Consider the many depictions of personal loss-related resentment or aggression in myth or folklore. In the Korean song *Arirang*, which became popular as a resistance anthem during the colonial era (Atkins, 2007), the *haan* of an abandoned maiden leads her to spitefully prophesy that foot pain will befall her lover as he leaves her. The neuropeptide dynamics might differ only by degree from Achilles of Ancient Greece, who avenges the death of his best friend Patroclus by slaying Hector and dragging him behind his chariot; or Queen Clytemnestra, who murders her husband Agamemnon as retribution for the sacrifice of her daughter Iphigenia. In any of these actors, one can imagine the baseline of *jeong* (rising endorphins and enkephalins); and then as *jeong* dropped, the emergence of original *haan* (their attenuation); and then a transformation to constructed *haan* (dynamic modulation of vasopressin).^4^ More attention to the phenomenology of bondedness—enhanced, possibly, through appreciation of the arts and literature—could point toward testable scientific hypotheses about the roles of neuropeptides and other mechanisms.

Importantly, though, we emphasize that a patriarchal societal framework is a likely backdrop to the constructed *haan* seen in many artistic representations of female agitation or aggression. For women in such situations, the consequences of a lost bond—specifically with a man upon whom they may be forcibly dependent—could be socially, economically, and bodily devastating. If constructed *haan* emerges through individual cognitive appraisal, then those appraisals are also influenced by prevailing norms. In contexts where male aggression is permitted, lauded, or attributed to biology, and female aggression may be consigned to mental instability, the real source of these interpretations may be the projection of societal expectations (*viz.*, Eurocentric schema, the WEIRD mind) onto the study of basic biological systems. For a contrasting example, the matriarchal Iroquois Confederacy of indigenous North Americans was a society of *male aggression* and *female power* (Prezzano, 1997), and our larger point is to encourage reflection on the embeddedness of all behaviors. Hypotheses about “natural aggression” may derive from constructed cultural sources that have little or no foundation in mammalian neurobiology.

Further, consider that the weakening or ending of a social bond—or, conceivably, any relative deficit of *jeong* in one’s baseline state—may be an unobserved driver for vulnerable individuals to hurt themselves, to increase endogenous opioid activity. Numerous studies have reported lower levels of beta endorphins in the blood, cerebrospinal fluid, and saliva of patients who demonstrate non-suicidal self-injury (van der Venne et al., 2021; Störkel et al., 2021; Cakin Memik et al., 2023; Jackson et al., 2023). Although a recent prospective study did not show a correlation between plasma endorphins and likelihood to self-injure (Kao et al., 2024), an ecological study showed lower salivary endorphin levels in the moments just before a self-injurious act (Störkel et al., 2021) when endorphins were measured every two hours. These data are consistent with our proposal that the feeling of *haan*, including a propensity for aggression which could be turned inward on oneself, may be correlated with a relative deficit in endogenous opioid activity.

## 4 Case study: the *jeong* and *haan* of Vincent van Gogh

### 4.1 The stable euphoria of creative flow

In Section 3.1 we already noted how original *haan* can be an impetus for creativity, in that a sense of missingness or “unfinished business”—regarding a personal relationship, a soup simmering on the stove, an aesthetic perception, a scientific theory, or anything else—may be the driver for some kind of action to “complete” that which one perceives as “uncompleted.” In this section we aim to deepen the grasp of the feelings of *jeong* and *haan* by directing our attention toward the subjective experience of artistic creativity. While the essence of art may be such that it defies any single definition, most artists would probably agree that their creative process is intimately related to the state of *flow* as described by the psychologist Mihaly Csikszentmihalyi:

Artists, athletes, composers, dancers, scientists, and people from all walks of life, when they describe how it feels when they are doing something that is worth doing for its own sake, use terms that are interchangeable in their minutest details. This unanimity suggests that order in consciousness produces a very specific experiential state, so desirable that one wishes to replicate it as often as possible. (Csikszentmihalyi, 1988)

The neuroscientific study of the flow state is still in its infancy. Rosen and colleagues recently reported that creative flow may entail a brain state of “less thinking,” in that real-time monitoring of jazz musicians showed decreased activity in regions of the default mode network when they were in high flow (Rosen et al., 2024). Interestingly, they found gamma-band clusters in the left parietal and central opercula—regions of high opioid receptor binding (Baumgärtner et al., 2006). One common-sensical intuition is that flow—like brain-induced analgesia, maintenance of social bonds, and modulation of reward experience—arises in conjunction with elevations of endorphins and enkephalins.

**Figure 4:**
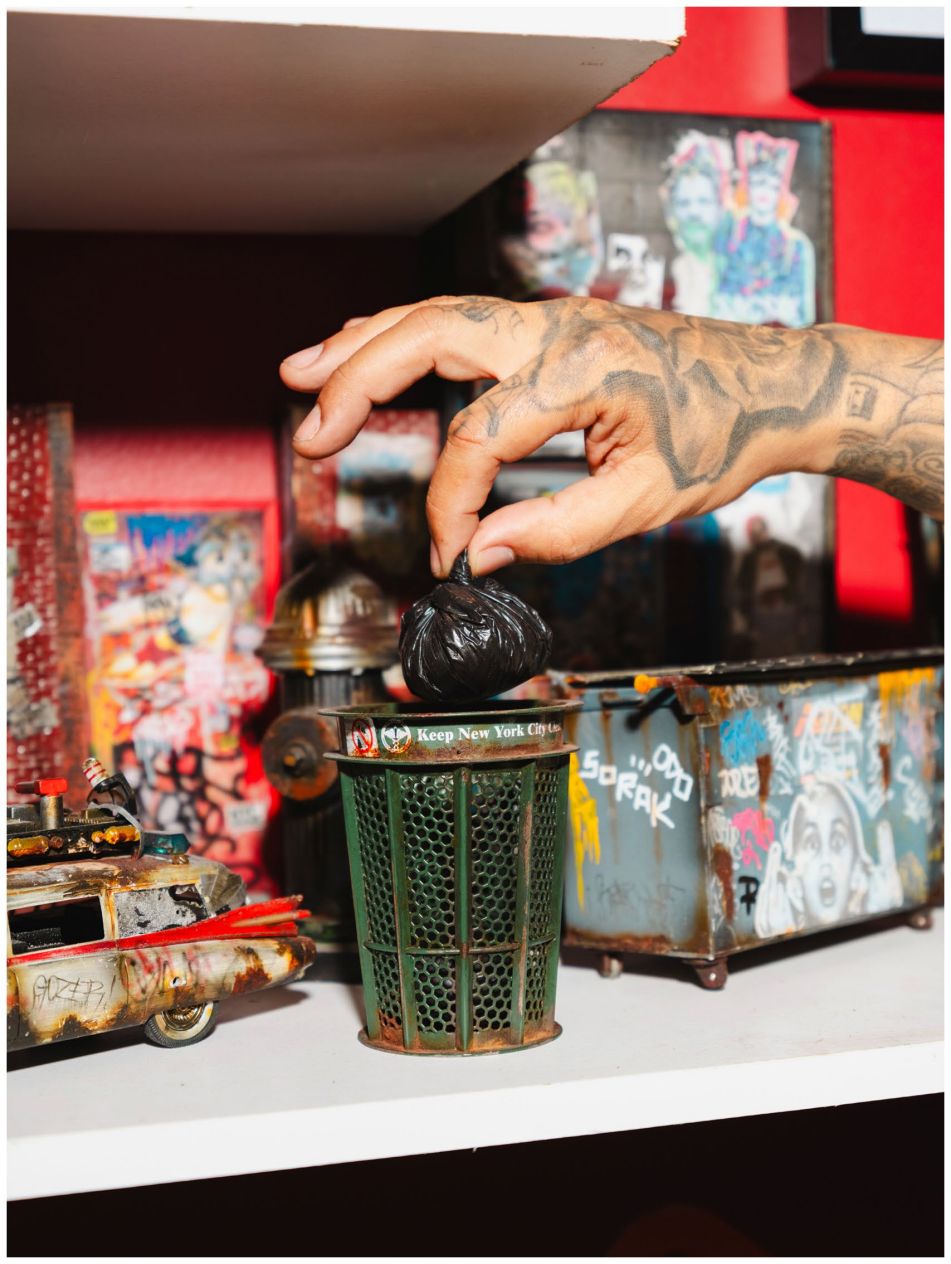
Miniature garbage can by Danny Cortes (Copyright Lanna Apisukh/The New York Times/Redux, used with permission).

Consider the story of the self-taught artist Danny Cortes. In sharing with a journalist about the origins of his work, Cortes told of how during the pandemic, he had been jobless, divorced, and on probation for selling drugs. His life was at “rock bottom.” Then one day, he discovered the hobby of constructing intricate dioramas using everyday household materials. He began to devote himself to recreating *jeong-*ful objects such as the bodega ice carts or graffiti-covered garbage cans (Figure 4) of his Brooklyn life. His account exemplifies the joy of creative flow:

I loved that when I worked on a piece, I did not think about my problems*—*my divorce, the pandemic… It was an escape*—*like I’m meditating, literally floating. I did not have a problem in the world. I wanted that high again, I kept chasing that. (Weisstuch, 2023)

Cortes further reminds us that beauty bypasses preconceptions. “I love everything abandoned… everything rusty, dirty. When you pass by a dumpster, most people usually do not take time to stop, breathe, forget about your daily life in New York and the hustle and bustle. Take your time, look around, you can see beauty in a rust drip.” Similar qualitative statements from creators of all kinds and in any field point toward another property of endogenous brain opioids that deserves its own framework: the Brain Opioid Theory of Stable Euphoric Creativity, or the BOTSEC.

Through his life story, inspirations and work products, Cortes illustrates how *jeong* and *haan* can be the motivation for artistic practice. One can feel *haan* in Cortes’s description of his despair and alienation during the pandemic, and his awakening to his *jeong* for the familiar and trusted objects of his neighborhood, which could be deepened by interpreting them in aesthetic fashion. And by embarking on those renderings—engaging in the active work of artistic creation—he entered a state of flow which is so attractive that he describes it in euphoric terms. And thus *jeong* may serve as a foundation for flow states which, too, entail brain opioid modulation. Pleasant or even blissful feelings may arise, conjointly, from *jeong* and creative flow, and may become mutually reinforcing. Furthermore, flow states are not necessarily “creative” in any artistic sense, in that one can be in flow from simply washing the dishes or combing a dog’s hair. *And not only are these* *flow* *states pleasurable, they may serve as a buffer against moments of vulnerability due to pain or weakened social bonds*. Conversely, being out of flow—including periods of artistic withdrawal—may exacerbate social rejection and physical pain perceptions.

Shared endogenous opioid mechanisms may be one of the biological mechanisms underlying the catharsis or emotional release that can be entailed by creative arts expression, and for why creative arts engagement can be a powerful tool for connection, community building and therapeutic interventions. Furthermore, through the neuroplasticity that comes with experience and learning, the practice of repeated arts engagement and, in turn, the honing of one’s art appreciation, could serve to enhance the brain’s sensitivity to pleasure derived from sensory experiences, and possibly even heighten the sensitivity to sensation itself.^5^ At the cellular level, again consider that opioids act as neuromodulators that decrease short-term neurotransmitter release. Conceivably they support complex integration and utilization of a wider *range* of signals related to aesthetics and social affiliation, given the role of opioids and their receptors as modulators of reward rather than for the direct feeling of reward. That is to say, they are like the set crew of a theater, adjusting the tone and impact of a stimulus by calibrating lighting, music, and props so as to convey and highlight meanings. By “setting the stage” opioids are thus powerful *facilitators* of emotional and motivational states, helping to fine-tune awareness, thoughts and perceptual patterns, and behavioral output. We propose that examination of the interrelationships between the experience of flow, the creative arts and “sensitization of the senses,” and the sense of *jeong*, and the likelihood that they all at least partially depend on endogenous brain opioid activity, is an important future direction for neuroaesthetics research (Chatterjee and Vartanian, 2014; Iigaya et al., 2020).

### 4.2 Why did Vincent van Gogh hurt himself?

In this section we aim to illuminate one of the most notorious incidents in the history of modern art, Vincent van Gogh’s self-severing of his ear, through the perspective of *jeong*, *haan*, and euphoric creativity, along with the neuropeptide dynamics discussed throughout this paper.

Psychologists, art historians, and amateur sleuths have not given up on attempting to explain, once and for all, why Van Gogh mutilated himself. Runyan (1981) summarized 13 different theories for the act which ranged from frustrated sexual drives to the influence of watching bullfights in Arles where the matador was given the ear of the bull as a reward. More recently, Murphy (2016) combed hospital and police records and even interviewed descendants of Vincent’s acquaintances, to theorize that he was moved to present the gift of his ear to a humble cleaning woman—not a prostitute, as legend has it—out of a tender desire to give of his own body, to help her heal an injury from a dog bite. There is so much mystery around Van Gogh’s ear that two art historians have even claimed that his friend Paul Gauguin detached it through an absurd feat of swordsmanship, and that the men made a pact not to tell anyone (Van Gogh’s ear/the pact of silence, 2008).

We will never know the exact ideations, feelings, and/or hallucinations that preoccupied Vincent van Gogh on the day of this harm which, despite any suspicion cast on Gauguin, surely happened by his own hand. Prior to the incident of late 1888, Van Gogh had already shown himself capable of self-injury. Once, while demanding to see his cousin whom he had asked to marry—she rejected him—Vincent “thrust his hand over a lamp, and refused to remove it from the open flame, begging her dumbfounded parents to let him see her” (Murphy, 2016). What we aim to add is a more nuanced awareness of the *intense and rapid swings in his perception of bondedness and loss*—his fluctuations of *jeong* and constructed *haan*—which likely led to acute and severe dysphoria. In the following paragraphs we share a granular view of the *unstable euphoria* he may have felt in the months before his self-injury, and we speculate about the neuropeptide dynamics that may have mediated changes in mood and outlook that culminated in his hurting himself.

Helped by his brother Theo, in 1888 Vincent made an arrangement with Gauguin for the two to paint together in Arles. To Van Gogh, the prospect of connecting—becoming *jeong-*ful—with Gauguin was deeply exciting. Throughout his life, Van Gogh was poor and largely unrecognized, while Gauguin was a rising star in Parisian art circles—older, and experienced from exotic travels. Vincent created a studio for them with hopes of it leading to a colony, and that summer he painted some of his greatest pieces. His sense of *jeong* was already building: for the new home, the new region and its beautiful scenery, and anticipation of his new partner in art.

**Figure 5:**
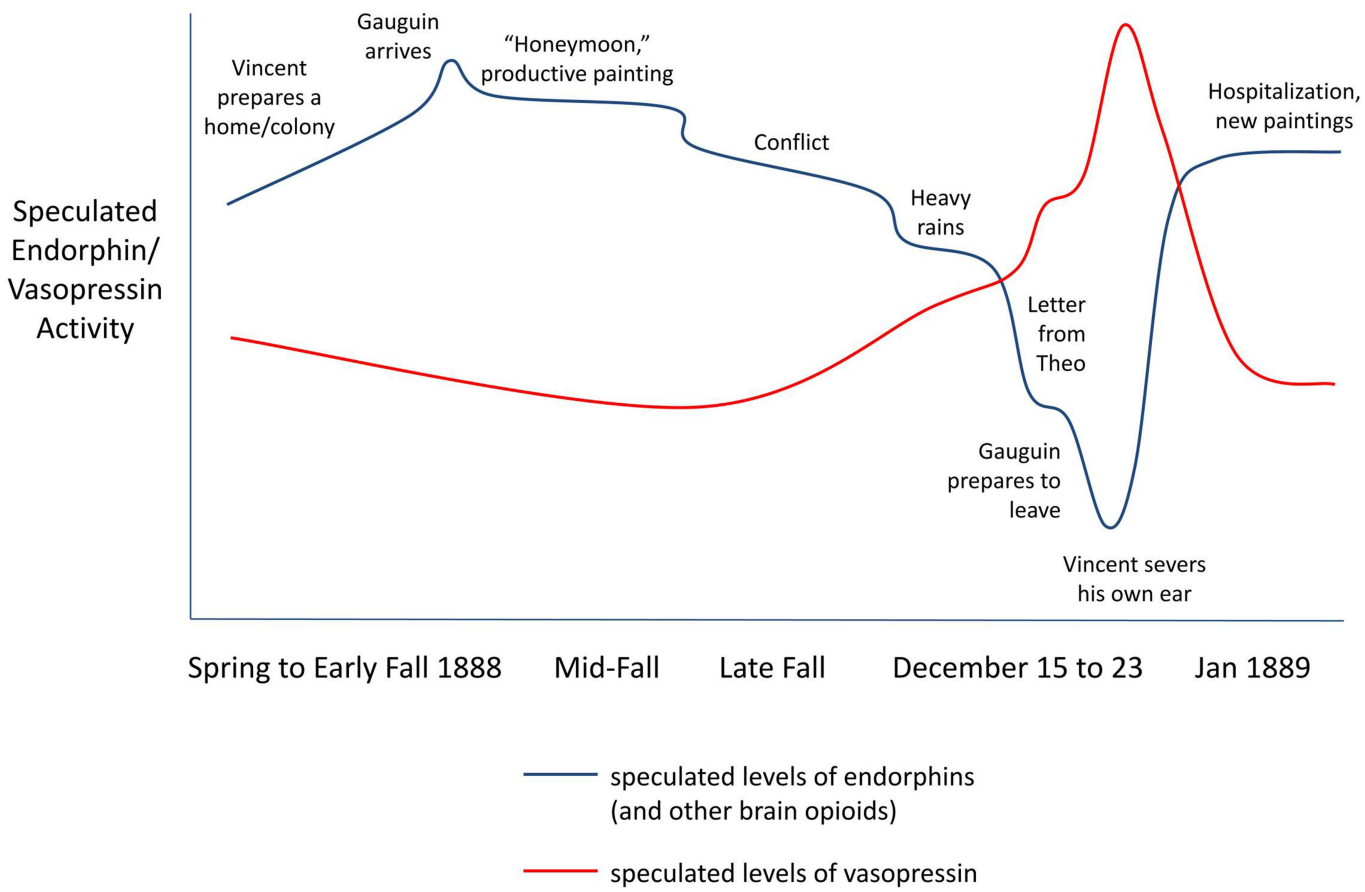
Speculated activity of brain opioids and vasopressin influencing Vincent van Gogh before and after his self-injury, when he was eagerly awaiting Paul Gauguin’s arrival, during their 2 months living and painting together, and his post-injury hospitalization. Brain opioid and vasopressin activity levels are predicted by the brain opioid theory of social attachment (BOTSA), the brain opioid theory of stable euphoric creativity (BOTSEC), and the brain opioid and vasopressin theory of original and constructed *haan* (BOVTOCH) as well as data showing that physical warmth facilitates social warmth through a brain opioid mechanism. Degrees of change are exaggerated for illustrative purposes.

When Gauguin finally arrived, brain opioids crescendoed, and Vincent was thrilled. Gauguin helped bring more order to the place—he was a good cook—and for a while they were both happy. They probably both experienced prolonged states of creative flow, mediated by endogenous opioids. Yet soon there was tension. While he had great respect for his elder, and Vincent benefited for example from Gauguin’s guidance to paint from the imagination, Van Gogh was opinionated, stubborn and righteous. They quarreled sharply. Moreover Vincent seemingly was unable to give his friend space; toward the end of their time together, he would even hover over Gauguin’s bed at night. The more he wanted, the less the Frenchman was willing to give. With social rejection, endogenous opioid activity in Vincent’s limbic and reward regions potentially destabilized and began falling (Figure 5), and possibly to precipitous levels.

It was not long before Gauguin had had enough, and Van Gogh felt betrayal as he feared his friend’s departure, with dread at the intensification of the all-too-familiar hole of emptiness that would follow the emotional highs of his relationships. Heavy rains began December 15—and intriguingly the affiliative effects of endogenous opioids are at least partially dependent on body temperature, such that physical warmth facilitates social warmth (Inagaki et al., 2015). For Vincent the perfect storm crested on December 23rd, when he opened a letter which, by some accounts, was from Theo, telling of his engagement to be married. Vincent fell into an abyss, blaming Gauguin for his misery. The BOVTOCH predicts a consequent rise in vasopressin, promoting aggressive actions that could give more control over the relationship. He called out the Frenchman for his despicable gall at daring to abandon him, and brandished a razor, causing Gauguin to flee. Perhaps his strong moral instinct—Van Gogh was the son of a minister—prevented him from threatening Gauguin further. Later that day, though, the toxic *haan* at Gauguin’s treachery finally overcame him. Vincent sliced off his ear, wrapped it, and presented it to a woman who worked at a brothel.

**Figure 6:**
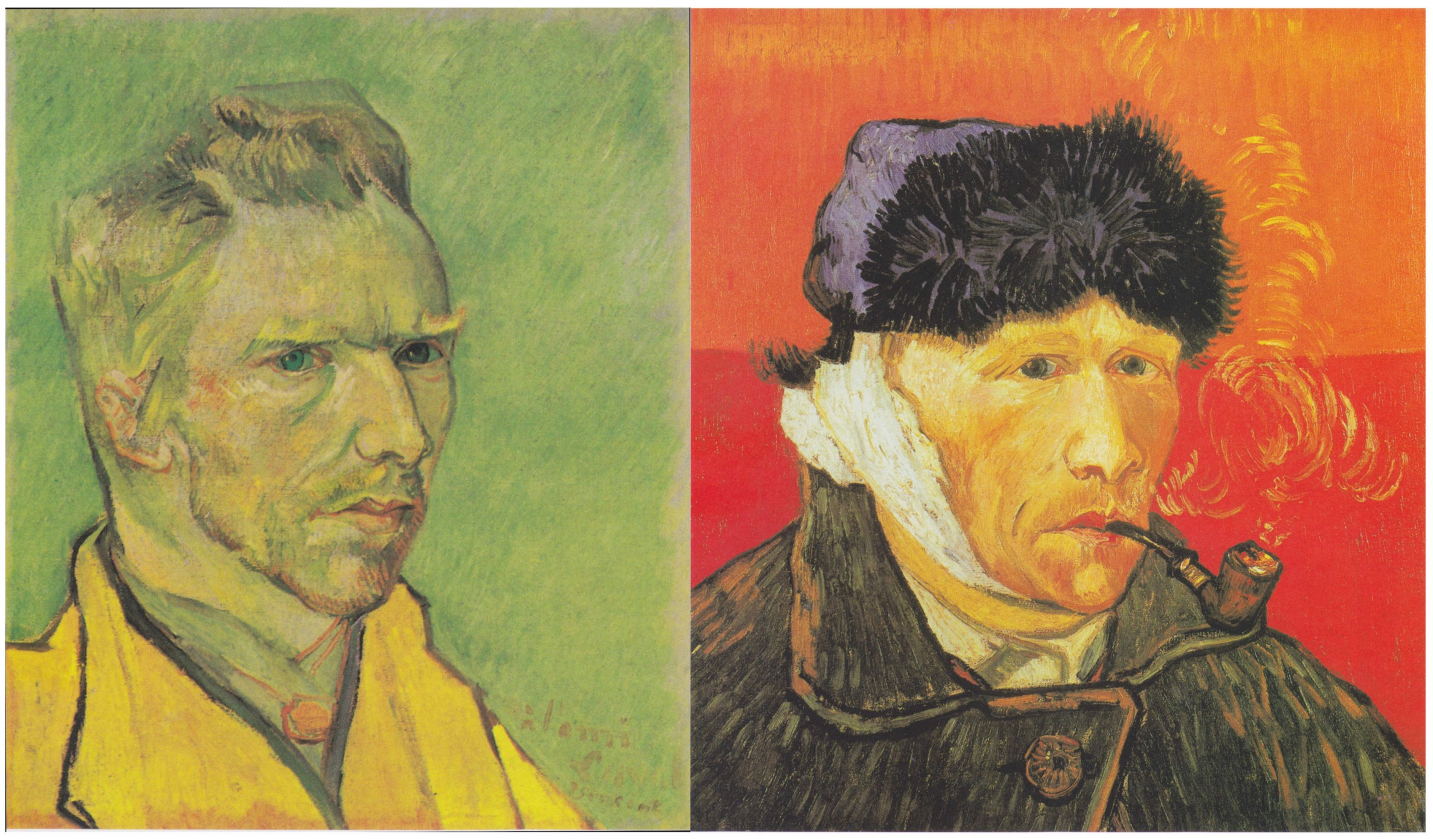
Van Gogh’s self portraits in November/December 1888 (left), while he was living with Paul Gauguin and quarreling with him; and in January 1889 (right), during his hospitalization after his self-injury. The latter rendering evokes a greater sense of calm and contentment, which we speculate was mediated by elevated endogenous opioid activity from brain-orchestrated analgesia, the *jeong* he felt from the hospital staff and gardens, and his flow of creative output.

Van Gogh’s hospital self portraits still suggest loneliness (Figure 6), yet they are remarkable for their calmness, and he wrote at the time that he did not feel “mad.” He was certainly benefiting from the *jeong* of the staff and the peaceful hospital gardens. Moreover his stay in the asylum at Saint-Rémy was the beginning of a period of phenomenal productivity—the bulk of his output was in 1889 and the following year—that raises the unsettling possibility that, in mutilating himself, Van Gogh had found a way to enhance and stabilize his endogenous opioidergic tone. In May of 1990 Van Gogh left to be with some artists in Auvers, near Paris, yet he was homesick for the Netherlands, and he also knew that his situation posed a financial strain on Theo, who by then had an infant son. Though Van Gogh was under the care of a sympathetic physician, he “could not bear to be left alone” (Estiene, 1972), and Dr. Gachet had other duties that required him to be away for a few days each week. In June 1890, the monthly allowance from Theo did not arrive. Seemingly, Vincent suffered another intense withdrawal from his own brain opioids later that summer, so that he could think of no other way to overcome his emptiness than to end his life.

**Figure 7:**
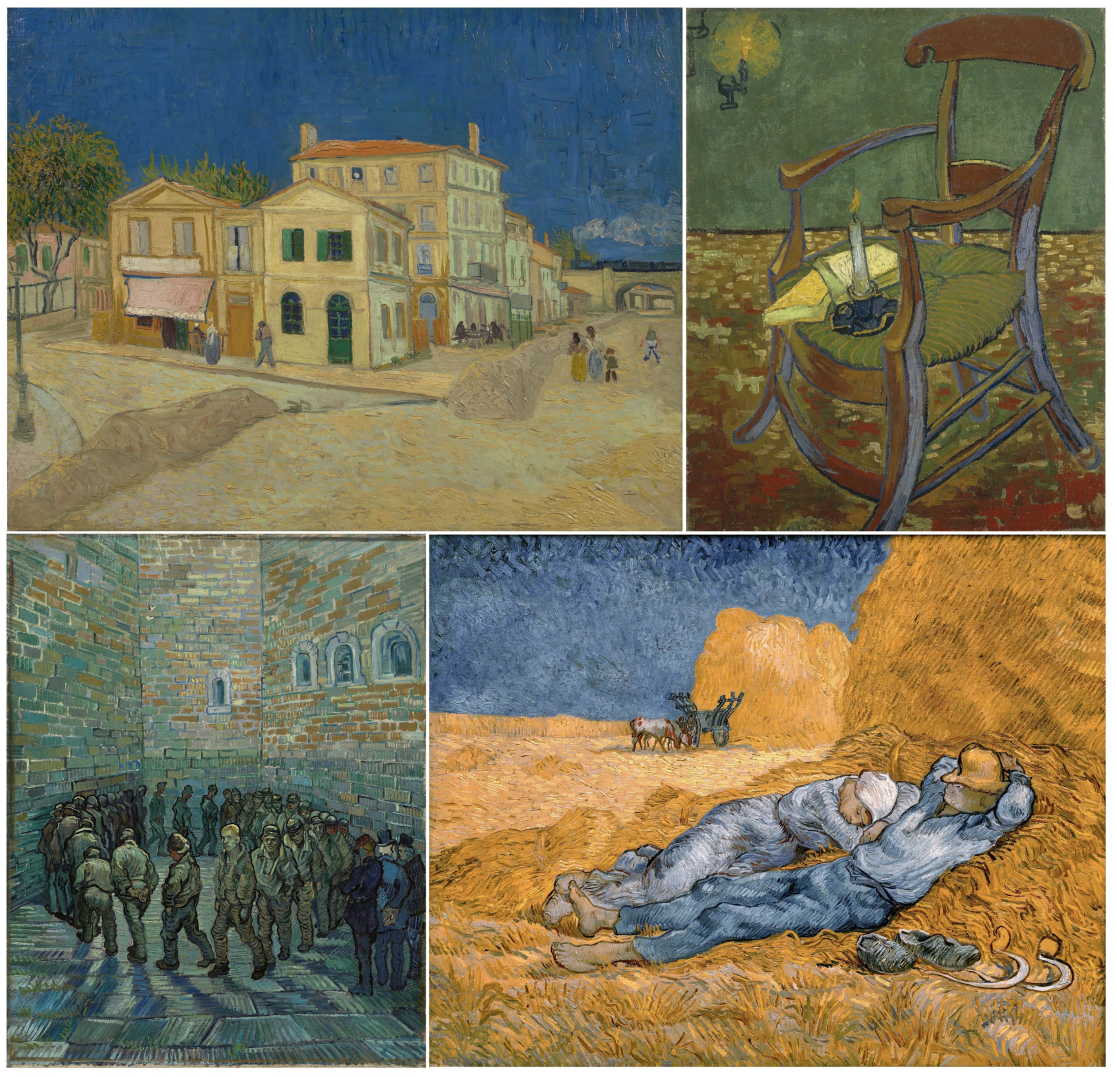
*Jeong* and *haan* illustrated in Van Gogh’s art. “Yellow House” (1888, upper left) in Arles, may be a reference to “house of friendship” in Japanese culture (Wallace, 1969). In “Gauguin’s Chair” (1888, upper right), one can feel Vincent’s resentment of his friend’s passionate enjoyment of nightlife. “Noon Rest from Work” (1890, bottom right) shows kinship not only between two humans, also the paired shoes, scythes, hay bales, even the oxen and features of the cart in the remote distance. “Prisoners’ Round” (1890, bottom left), after a print by Gustave Dore, shows deep despair yet can not hide Van Gogh’s appreciation of the warmth in all life.

*Jeong* and original *haan* are also palpable in Van Gogh’s art (Figure 7), and in more than one way. There is a visceral sense of connectedness (or missingness) in his deeply empathic portrayals of people and objects, in the unexpected harmony of contrasting colors, and even in how he preferred to display his paintings so that they would be “in conversation” with one another.^6^ Conceivably, Vincent van Gogh’s sensitivity to new and multifaceted ways of feeling - and expressing - *jeong* and *haan* has been a reason for his enduring appeal.

## 5 Discussion

We opened this paper with a remark from Bertrand Russell, who observed that for more than two thousand years the West has philosophized about either the tightening or relaxation of social bonds. We have aimed to extend that conversation by advancing neurobiological insight around the felt essence of bondedness. We then highlighted the thesis that the modern (or WEIRD) mind traces to a papal agenda in early Christian Europe to prohibit close-cousin marriages, and that a consequence was a weakening in the power of *kinship* bonds as glue for societal structure. What emerged was an individualistic and “impersonally pro-social” psychology which entailed greater participation in the power of *freedom* as well as “success” at a civilizational scale. We also pointed out that at least some behavioral scientists who study *attachment* think that there is inadequate language (in English) to discuss the feeling or phenomenon of *bondedness*, and that these two large-scale observations are coherent with one another. If both are substantially correct, then we hope that this paper may help overcome gaps of understanding of *Homo sapien* minds that maintain a high level of attunement (for better or worse) to ultra-fine gradations in kinship bonds, and that it might even be able to comment on aspects of the modern condition that are common in more atomized Western nations yet are—given globalization—hardly limited to them.

Potentially, *jeong* and *haan*, two words and feeling states derived from Korean yet in our usage not delimited to any culture or even to humans, may be useful as linguistic expressions for a contented and vaguely visceral sense of connectedness; and for the sense of loss, missingness, or “unfinished business.” The latter subdivides into what we call original *haan*, a feeling that is preverbal and especially subtle, and a constructed form of *haan* that arises as resentment, grievance, or prolonged sorrow using mechanisms of narrative and cognitive appraisals. We hope these words—and, more importantly, the feelings represented—may enter scientific and cultural lexicon and help advance the capacity for context-sensitive or relational (Oliva and Torralba, 2007) perception.

Attention to *jeong* (endorphins and enkephalins) and *haan* (diminishment of opioids, with or without a rise in vasopressin) could potentially fill gaps in study of a range of topics in behavior and health. We already noted how the proposed neurobiology of *haan* points toward the recovery of *jeong* as a way to overcome aggression (Section 3.3). Among other phenomena or endeavors that might benefit from BOTSA, BOVTOCH, or BOTSEC-related insights, there is play *as such*, which can enhance bondedness (Colonnello et al., 2011); variation in culture-driven behaviors, for example if immigrants maintain strongly kinship-focused belief systems (Mandavia et al., 2017); the role of art as a way to develop empathy (Peloquin, 1996); child development and educational methods that may facilitate healthy trajectories for bond-related feelings (Gerdes et al., 2015); mental health practice at large, for example in relation to the therapeutic alliance (Bar-Kalifa et al., 2019); companionship with animals (Hui Gan et al., 2020); or, what appears to be bonding or attachment between humans and human-engineered generative AI-based chatbots.

To close this paper, we speculate how the interrelated theories of endogenous brain opioid activity that we have presented could help to illuminate three large-scale phenomena of public health or healthcare. Rather than focus on specific testable hypotheses that would vary based on subspecialty interests, our aim is to show how incorporation of the social feelings of bondedness and loss—and their likely neuropeptide mechanisms—could inform a range of investigations.

“Here was a panacea for all human woes, here was the secret to happiness” wrote Thomas De Quincey (1821) in *Confessions of an English Opium Eater* regarding the euphoric and pain alleviating powers of laudanum, a popular tincture of the Victorian era. Introduced as a medical nostrum in the 1500’s, laudanum was composed of alcohol and dried extracts from the opium poppy that contained both morphine and codeine alkaloids, and it was only one in a long line of opioid substances that have led to the sickness of addictions over centuries and across the globe.

Today’s opioid addiction crisis has stemmed from inappropriate promotion of opioid analgesics by pharmaceutical companies, changing medical attitudes toward pain management (partly encouraged by drug company marketing), and a deregulated environment, nonetheless the BOTSA raises the hypothesis that any chronic usage of exogenous opioid drugs, whether prescribed or illegally acquired, may at least *partly* reflect lower endogenous activity which would otherwise be generated by the feeling of social bonds (Christie, 2021). Both cross-sectional (Yang et al., 1982a) and longitudinal analyses (Yang et al., 1982b) have reported that social isolation is associated with an opioid use disorder among older adults in the US. We consider the opioid epidemic to be *prima facie* evidence of fraying in the sense of connectedness—in conjunction with numerous other factors—leading to a state of vulnerability. This hypothesis is consistent with the thesis of the economists Anne Case and Angus Deaton, that the opioid crisis and other “deaths of despair” that increased among rural white American populations beginning in the 1990s have stemmed from loss of social cohesion in the aftermath of degradation of the manufacturing sector (Case and Deaton, 2020).

Nearly four decades of basic science research have demonstrated a powerfully modulatory role of environmental enrichment as well as affiliative and antagonistic social interaction on drug taking, drug seeking, relapse and reinstatement (Levis et al., 2021). Laboratory animals who are socially isolated or have been exposed to aversive social interactions—where endogenous opioid tone may be diminished— self-administer opioid drugs or psychostimulants at higher levels than animals who are group housed or exposed to positive social interactions (Vivian and Miczek, 1999; Venniro et al., 2018; Venniro et al., 2019; Venniro et al., 2021). In the pioneering “rat park” study, rats living in social isolation, as opposed to large housing, preferred drinking a sweetened morphine solution over water. In group-housed rats, this preference was reversed (Alexander et al., 1978; Alexander and Hadaway, 1982). In operant social choice models, methamphetamine, heroin, or cocaine addicted mice were found to robustly prefer social interaction over drugs (Venniro et al., 2018; Venniro et al., 2019). Numerous reviews of the influence of social factors in animal models of addiction are available (Mason et al., 1963; Lu et al., 2003; Levis et al., 2021; Malone et al., 2022; Venniro et al., 2022; Marchant et al., 2023). Social theories of opioidergic tone may help to explain the success of community-reinforcement approaches to addiction treatment (e.g., Alcoholics Anonymous), whereby individuals learn to replace drug use with nondrug social rewards that are contingent on cessation of drug use (Hunt and Azrin, 1973).

Secondly, social connectedness influences health directly. As we noted in Section 2.3, pain is one example, where many studies have shown relationships between social or physical pain and endogenous opioid activity (Hsu et al., 2015; Sullivan and Ballantyne, 2021; Dueñas et al., 2016; Ren et al., 2021; Margari et al., 2014; Timm et al., 2023; Johnson and Dunbar, 2016; Khosravi et al., 2021; Manninen et al., 2017; Martin et al., 2015; Noguchi et al., 2023). These mechanisms can create a risk for vicious cycles; for example, analysis of longitudinal data from England found that loneliness was a risk factor for back pain at a later time, and back pain was a risk factor for worsening loneliness (Suzuki et al., 2024).

Even more broadly, there is now copious evidence that a lower level of social connectedness confers a risk for a range of physical diseases and earlier mortality. The overall magnitude of the effect is comparable to or greater than cigarette smoking, high blood pressure, physical inactivity and other known modifiable risk factors (Holt-Lunstad, 2022), and it has led the US Surgeon General to describe loneliness as an epidemic and public health threat (Harris, 2023). To be sure, these findings likely result from multiple mechanisms such as neuroendocrine dysregulation, excess sympathetic activity, and other processes that accumulate to increase “wear and tear” or allostatic load on the brain and body (McEwen, 2007). Yet consider that mu opioid receptors (and others in the G protein-coupled class) can be understood as ubiquitous regulators of the orchestrated interface between health and disease (Leysen et al., 2021). Beta endorphins modulate both inflammation and oxidative stress (Pilozzi et al., 2020; Pandey et al., 2023). Conceivably, low levels of endogenous brain opioid activity stemming from prolonged states of poor social connectedness may be an upstream influence on morbidity and mortality. This idea could help explain the “French paradox” (Renaud and de Lorgeril, 1992)—the relatively low cardiac disease prevalence despite a diet high in saturated fats and cholesterol—if *fraternité* combined with the moment to moment *joie de vivre* held by many French to be a core national value can help to maintain a threshold level of opioidergic tone. Is the longevity of individuals in “Blue Zone” regions of the globe (Poulain et al., 2013; Poulain et al., 2021)—for example Okinawa or Sardinia—dependent on the strength and consistency of their community bonds? Intriguingly, one study of “super agers” with extraordinary episodic memory found that across six subscales of psychological well-being, they differed from their cognitively average peers in only their greater levels of “Positive Relations with Others” (Maher et al., 2017).

**Figure 8:**
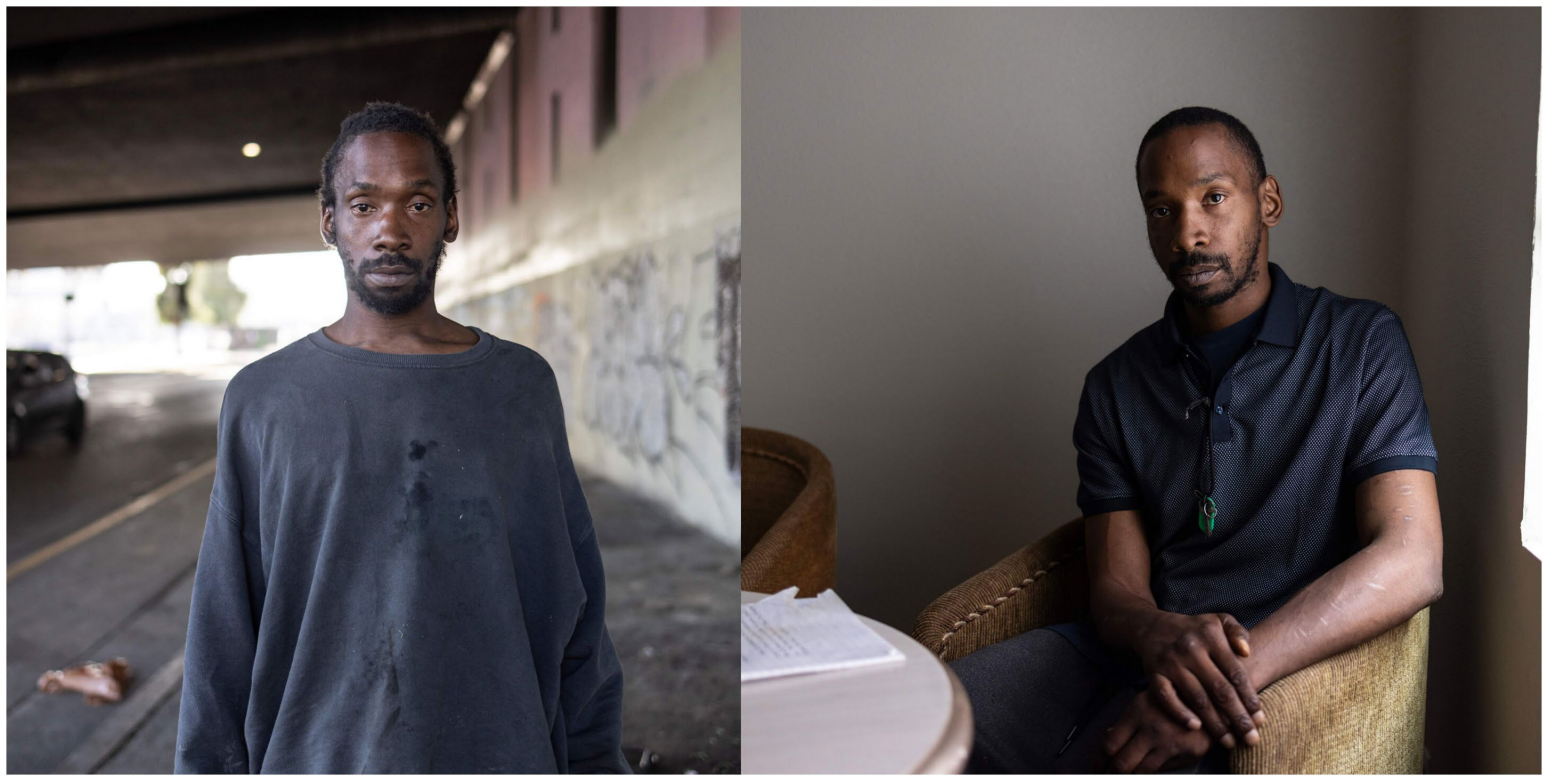
Photographs of a man before and after treatment through a “Street Psychiatry” program in Los Angeles (Copyright Rachel Bujalski/The New York Times/Redux, used with permission). Left, Yoh had been living under a freeway, and was visited by case workers for 5 months, who looked for him and brought bottles of water and hamburgers, thereby building trust and *jeong*. Eventually, Yoh agreed to begin oral and then injectable antipsychotic medications, and as his symptoms improved he moved into interim housing. A fingerprint database revealed his name to be Eric Covington (right), and he began to recall the life he had lived before his psychotic break. According to a journalistic account, “[p]erhaps the most striking change in Mr. Covington was that he showed a desire for human company.” For a contrary view which contends that “Housing First” is superior to “Treatment First” as a strategy against homelessness, see Footnote 7.

Or, consider an aspect of a modern development in medical care in context of the homelessness crisis, one which seemingly illustrates the power of *jeong* in supporting the most vulnerable. Sophisticated psychopharmacological treatment programs have recently emerged for treating unsheltered homeless people (Bromley et al., 2024) and in some cases helping them to transition toward stable shelter and reclaiming their lives. One journalistic profile of a “Street Psychiatry” initiative (Barry and Bujalski, 2024) illustrated how, in conjunction with overcoming many hurdles, a thoughtful process of care and respect for clients to include, seemingly, forming a *bond* with them—through multiple visits to gain trust, patiently looking around tents, conducting seated interviews in parking lots, carefully intuiting whether explanations are being understood—is critical to engaging them in the care they need for recovery, including scheduled use of antipsychotic medications (Figure 8). In addition to physical shelter, an authentic sense of connectedness is a fundamental human need, and perhaps—at least in some cases—the latter may be a priority for the former to be relevant.^7^

As a converse to the possible effects of *jeong* on health or medical care, the BOVTOCH could help to illuminate why hostility is a risk factor for incident cardiac disease (Chida and Steptoe, 2009). In Section 3.3 we described vasopressin as an ancient “fight-or-flight” molecule whose role for raising blood pressure and conserving water—again, it is also known as antidiuretic hormone—addresses the need to divert blood toward large muscles in a setting of danger, and to prevent loss of circulating volume in case there should be penetrating injury or if water is perceived to be scarce. To the degree that vasopressin levels may be elevated in patients with heart disorders, one can hypothesize a sequence from the loss of *jeong*, to constructed *haan*, to heightened sympathetic activity and higher likelihood for coronary inflammation, and prothrombotic states—functions that all relate to aggression and the potential consequences of penetrating physical trauma—and one cross-sectional Chinese study has found that adults with hypertension had higher vasopressin and lower beta endorphin levels (Zheng et al., 1995). While there is likely mutual reinforcement of hostile temperament and social isolation, raising questions of “chicken or egg” priority, the BOVTOCH suggests that isolation comes first—endorphin withdrawal disinhibits vasopressin—and an implication is that enhancing and maintaining the opioid-mediated feeling of connectedness may be key to the remediation or ultimate healing of any aggressive condition. Future studies of health and aging may profit from more attention to bondedness or *jeong* and *haan*-related variables across scales, from communities to behaviors, brains, or molecules, to identify critical factors or sensitive periods, and to evaluate interventions that are attentive to these mechanisms.

Third, the theories we have presented may help to further ground understanding of the placebo response, or effect. The placebo *response* has been defined as physiological change that can be traced to subjective expectations, associative conditioning (for example chronic use of a medication that leads to Pavlovian changes), and “doctor-patient communication” (without necessarily recognizing the independent influences of the doctor-patient *relationship*); and by contrast the placebo *effect* may include placebo responses yet also encompass natural history of disease, regression to the mean, reporting bias, or non-specific co-interventions (Schedlowski et al., 2015). The very need to articulate this distinction indicates that modern biomedicine still has only a tenuous grasp on the subjectivity of healing processes, or attributions around the “causes” of health (Lee, 2019), and a stronger appreciation for social bond-related endogenous opioid activity may be necessary.

To be clear, the placebo response is a “good” thing if its elicitation is safe and ethical. Yet while saline injections may promote greater mu opioid receptor activity when described in salutary language in a setting of pain (Zubieta et al., 2005), if placebo research focuses too narrowly on the analgesic properties of endorphins or enkephalins then it may overlook the subtleties of opioidergic tone (BOTSA) that we theorize to depend on *jeong*. We contend that advanced understanding of placebo-related phenomena will require greater recognition of the many roles played by neuropeptides. Specifically, we propose that healthy leveraging of the placebo response may be as reliant on the *quality* of the connection between a caregiver and a patient or research subject—that is to say, the character and the valuation of the *jeong*—as on any particular content of “communication.” If this proposition is correct, then “lack of *jeong*” could help to explain why the WEIRD mentality invented the “placebo” concept in the first place—to grasp at the hole that opened when medicine crossed a threshold from healing ritual that appreciates the subjectivity of health and recovery, to an objective science based on skepticism. In the United States, there is high dissatisfaction (BOVTOCH) with the healthcare system on the part of both patients (who may seek out alternatives) and providers (who are increasingly burned out, in some cases due to requirements to “treat” the electronic medical record), and it is undoubtedly also true that many care providers become stressed from being the only source for *jeong* that has eroded in homes and communities. Making these matters worse, the incentive structure of American healthcare economics downplays “soft” contributions to health or well-being, or the kinds of activities that are supportive of *jeong*. Case and Deaton have argued that it was not only the loss of good jobs that led to deaths of despair in rural America; a “rapacious” system prioritizes complex medical procedures and creates the net effect of transferring wages away from the working class (Case and Deaton, 2020).

**Figure 9:**
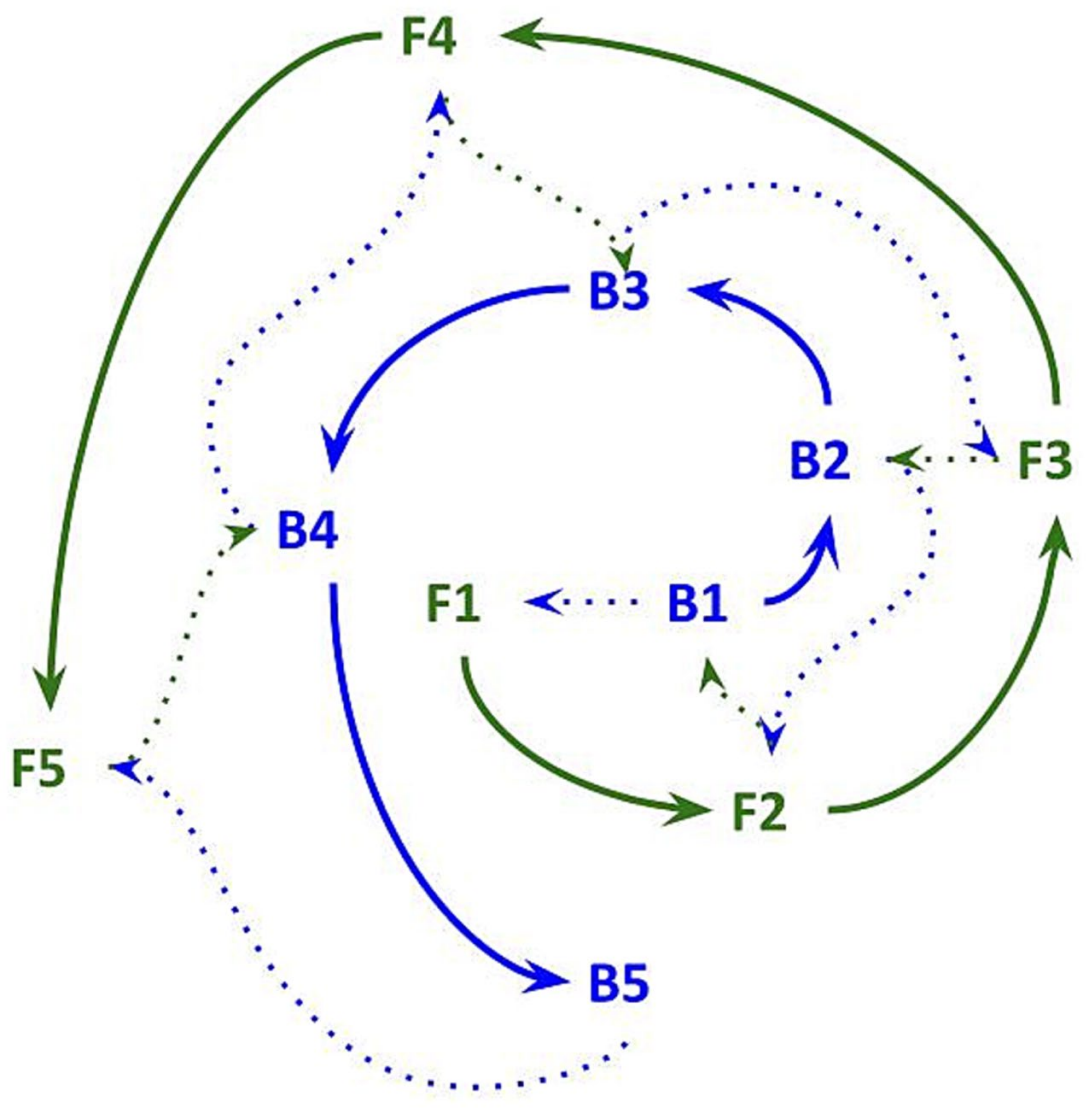
Virtuous cycles that leverage the shared opioidergic mechanisms of social bondedness and stable euphoric flow. A given *jeong*-ful bond (B1) will enhance the euphoric flow (F1) of the bonded individuals so that their creativity takes on a new character (F2). Yet the new flow state (and the created product) reciprocally influence the bond itself to evolve (B2). The new state of bondedness influences their euphoric flow again (F3), and the cycle continues. Also note that the B and F indicators in the figure may be transposable. A state of flow might arise, for example, because two bonded individuals engage their flow together; alternatively an individual’s flow state might attract a connection and bonding with others who share a “similar flowing interest.”

In conclusion, we reiterate our earlier point (Section 4.1) that engaging in the arts and enjoyment of the flow state (Hinz et al., 2022) can enhance *jeong* or connectedness in a virtuous cycle of mutual positive reinforcement (Figure 9); and if arts or creativity interventions can help to let go of negative appraisals and blaming, we may avoid the hazards of constructed *haan*. The opportunity to create anything is after all always an opportunity to create *together*, so that social bonds and creative flow can mutually enhance one another. Such a dynamic powers musical bands, theater or dance companies, dyads of directors and actors, many scientific collaborations, and many companies, organizations, and spiritual communities, or any other group of creators that comes together, where members enjoy one another’s presence, and where they create things with one another.^8^

We hope this paper, and its three related theories regarding endogenous brain opioids, may thus further encourage creators to work in collaborative ways, and that it helps remind those who already create through ensembles that “all the resources they need” are already in their own brains—to modulate neurotransmission, and to “set the stage” for all salient signaling—such that exogenous opioids should be used, when necessary, only sparingly; and that it encourages use of original *haan* for evolving toward wholeness rather than negative forms of constructed feeling.

## Data Availability

The original contributions presented in the study are included in the article/supplementary material, further inquiries can be directed to the corresponding author/s.

## Footnotes

^1^To further illustrate how *jeong* does not require intimacy, consider two students seated next to each other in a classroom. After a few weeks, they may say that *jeong* has developed, even if there has been no physical contact (or even conversation). There is a long history of scholarly commentary on *jeong* (정/情) and *haan* (한/恨) as emotions that can not be separated from social virtue or spirituality. While modern Koreans may not agree on the role of these emotions in contemporary Korean culture (and see, especially, Section 3.2), our approach in this paper is to value the recognition of these emotions by describing them in naturalistic terms that may be salient for both social and affective neuroscience, and healthcare. See Chung and Oh (2022) for a recent volume of essays that consider *jeong* and *haan* from societal and cultural perspectives.

^2^Reportedly, the French mathematician Blaise Pascal found cycloid curves “so distractingly fascinating that he claimed merely thinking about them could relieve the pain of a bad toothache.” (Hart, 2023).

^3^In other words, we oppose the segregating idea that “only Koreans can feel *haan*, or that Korean culture is itself defined by *haan*.” One might state for example that the journey home by Odysseus after the Trojan War was motivated by deep original *haan*, and *haan* may be said to motivate any aggrieved person or culture. Nonetheless for geopolitical and historical reasons, both original and constructed *haan* may continue to be readily perceptible to Koreans, especially insofar as Confucian principles lend themselves to constructed moral appraisals, and also to the degree that Koreans feel the emotional “missingness” associated with national (peninsular) division, competition due to economic development based on capitalism; and as with many other modern nations, alienation from domestic polarization. While Han Kang’s writings may, for example, reflect the "death of *jeong*", her studied inattention to the feeling of connection is an emphatic reminder, through its absence, of its necessity. Kang’s writing is deeply engaged with original *haan*, even if she herself might reject such an assessment.

^4^In Korean folk psychology, obstruction or suppression of *haan* may lead to *hwa-byung* (화병) or “anger disease,” a condition that the *Diagnostic and Manual* has recognized as a “cultural” syndrome (Lee et al., 2014). *Hwa-byung* is often conceptualized as being due to accumulation or suppression of *haan*, and Min has contended that *hwa-byung* helps to inform the rationale for describing a new category of anger disorder (Min, 2008).

^5^Interestingly a pilot study of an arts curriculum for dermatology trainees, co-led by professional fine arts educators and based at a fine art museum, was associated with improvement in observational skills (Huang et al., 2016).

^6^Van Gogh’s emotional tenderness, especially in the last two years of his life, is the subject of Van Gogh: Poets and Lovers, a 200 year anniversary exhibition at the National Gallery of London (Labarge, 2024).

^7^Some contend that regarding the societal challenge of homelessness, there is a stronger evidence base for a strategy of “Housing First” over “Treatment First” (Peng et al., 2020). Theoretically, some differences in the success rate of the two strategies may depend on their attentiveness to *jeong* or connectedness-related objectives.

^8^The energy behind such virtuous cycles can also be described, in Korean, as *heung* (興) or “exhilaration or utmost joy.” *Heung* typically has the connotation of intense outward expression (e.g., late-night drinking while singing Karaoke, K-pop, etc.), and the Chinese pictograph for this word derives from the idea of many hands coming together to lift an object. Yet *heung* can also be conceptualized as the energy or emotion that actively *propels* the departure from *haan*, and speculatively it may entail admixture of opioidergic tone with more arousing neurotransmitter or neuromodulatory systems. As Ilyoon Park puts it (Park, 2022), “[there is] coupling of han and heung… when the despair situation of han needs a warm encouragement, it will couple with jeong. Likewise, when it needs cheering up, it will do with heung.”

## References

[ref1] Achterberg E. J. M. van Swieten M. M. H. Houwing D. J. Trezza V. Vanderschuren L. J. M. J. (2019). Opioid modulation of social play reward in juvenile rats. Neuropharmacology 159:107332. doi: 10.1016/j.neuropharm.2018.09.007 30218673

[ref3] Ainsworth M. S. Bowlby J. (1991). An ethological approach to personality development. Am. Psychol. 46, 333–341. doi: 10.1037/0003-066X.46.4.333, PMID: 28347152

[ref4] Albers H. E. (2012). The regulation of social recognition, social communication and aggression: vasopressin in the social behavior neural network. Horm. Behav. 61, 283–292. doi: 10.1016/j.yhbeh.2011.10.007, PMID: 22079778

[ref5] Alexander B. K. Coambs R. B. Hadaway P. F. (1978). The effect of housing and gender on morphine self-administration in rats. Psychopharmacology 58, 175–179. doi: 10.1007/BF00426903, PMID: 98787

[ref6] Alexander B. K. Hadaway P. F. (1982). Opiate addiction: the case for an adaptive orientation. Psychol. Bull. 92, 367–381. doi: 10.1037/0033-2909.92.2.367, PMID: 7146233

[ref7] Al-Hasani R. Bruchas M. R. (2011). Molecular mechanisms of opioid receptor-dependent signaling and behavior. Anesthesiology 115, 1363–1381. doi: 10.1097/ALN.0b013e318238bba6, PMID: 22020140 PMC3698859

[ref8] American Psychiatric Association (2013). Diagnostic and statistical manual of mental disorders. 5th Edn.

[ref9] Anthony Bourdain on Han: parts unknown (S. Korea). (2018). Available at: https://www.youtube.com/watch?v=VoDIkaDgaf4 (Accessed May 29, 2024).

[ref10] Ashok A. H. Myers J. Reis Marques T. Rabiner E. A. Howes O. D. (2019). Reduced mu opioid receptor availability in schizophrenia revealed with [11C]-carfentanil positron emission tomographic imaging. Nat. Commun. 10:4493. doi: 10.1038/s41467-019-12366-4, PMID: 31582737 PMC6776653

[ref11] Atkins E. T. (2007). The dual career of “Arirang”: the Korean resistance anthem that became a Japanese pop hit. J. Asian Stud. 66, 645–687. doi: 10.1017/S0021911807000927

[ref12] Aubry A. V. Joseph Burnett C. Goodwin N. L. Li L. Navarrete J. Zhang Y.. (2022). Sex differences in appetitive and reactive aggression. Neuropsychopharmacol. Off. Publ. Am. Coll. Neuropsychopharmacol. 47, 1746–1754. doi: 10.1038/s41386-022-01375-5, PMID: 35810200 PMC9372130

[ref13] Bar-Kalifa E. Prinz J. N. Atzil-Slonim D. Rubel J. A. Lutz W. Rafaeli E. (2019). Physiological synchrony and therapeutic alliance in an imagery-based treatment. J. Couns. Psychol. 66, 508–517. doi: 10.1037/cou0000358, PMID: 31144846

[ref14] Barry E Bujalski R. (2024). Under an L.A. freeway, a psychiatric rescue mission. The New York Times [Internet]. Available from: https://www.nytimes.com/2024/10/20/health/los-angeles-homeless-psychiatry.html (Accessed May 29, 2024).

[ref15] Bartz J. A. Zaki J. Bolger N. Ochsner K. N. (2011). Social effects of oxytocin in humans: context and person matter. Trends Cogn. Sci. 15, 301–309. doi: 10.1016/j.tics.2011.05.002, PMID: 21696997

[ref16] Baumgärtner U. Buchholz H. G. Bellosevich A. Magerl W. Siessmeier T. Rolke R.. (2006). High opiate receptor binding potential in the human lateral pain system. NeuroImage 30, 692–699. doi: 10.1016/j.neuroimage.2005.10.033, PMID: 16337817

[ref17] Becker J. A. J. Clesse D. Spiegelhalter C. Schwab Y. Le Merrer J. Kieffer B. L. (2014). Autistic-like syndrome in mu opioid receptor null mice is relieved by facilitated mGluR4 activity. Neuropsychopharmacol. Off. Publ. Am. Coll. Neuropsychopharmacol. 39, 2049–2060. doi: 10.1038/npp.2014.59, PMID: 24619243 PMC4104328

[ref18] Beery A. K. Lopez S. A. Blandino K. L. Lee N. S. Bourdon N. S. (2021). Social selectivity and social motivation in voles. eLife 10:e72684. doi: 10.7554/eLife.72684, PMID: 34726153 PMC8594915

[ref19] Berridge K. C. Robinson T. E. Aldridge J. W. (2009). Dissecting components of reward: “liking”, “wanting”, and learning. Curr. Opin. Pharmacol. 9, 65–73. doi: 10.1016/j.coph.2008.12.014, PMID: 19162544 PMC2756052

[ref20] Bodnar R. J. (2017). Endogenous opiates and behavior: 2015. Peptides 88, 126–188. doi: 10.1016/j.peptides.2016.12.004, PMID: 28012859

[ref21] Borland J. M. Walton J. C. Norvelle A. Grantham K. N. Aiani L. M. Larkin T. E.. (2020). Social experience and sex-dependent regulation of aggression in the lateral septum by extrasynaptic δGABAA receptors. Psychopharmacology 237, 329–344. doi: 10.1007/s00213-019-05368-z, PMID: 31691846 PMC7024004

[ref22] Born J. Lange T. Kern W. McGregor G. P. Bickel U. Fehm H. L. (2002). Sniffing neuropeptides: a transnasal approach to the human brain. Nat. Neurosci. 5, 514–516. doi: 10.1038/nn0602-849, PMID: 11992114

[ref23] Bromley E. Rahmanian Koushkaki S. Davis L. G. Moon S. Resnick J. McCoy M.. (2024). Addressing mental health disability in unsheltered homelessness: outpatient conservatorship in Los Angeles. Psychiatr Serv Wash DC. 75, 689–698. doi: 10.1176/appi.ps.20230235, PMID: 38268464

[ref24] Brown C. H. Scott V. Ludwig M. Leng G. Bourque C. W. (2007). Somatodendritic dynorphin release: orchestrating activity patterns of vasopressin neurons. Biochem. Soc. Trans. 35, 1236–1242. doi: 10.1042/BST0351236, PMID: 17956321

[ref25] Brownstein M. J. (1993). A brief history of opiates, opioid peptides, and opioid receptors. Proc. Natl. Acad. Sci. USA 90, 5391–5393. doi: 10.1073/pnas.90.12.5391, PMID: 8390660 PMC46725

[ref26] Buchel C. Miedl S. Sprenger C. (2018). Hedonic processing in humans is mediated by an opioidergic mechanism in a mesocorticolimbic system. eLife 7:e39648. doi: 10.7554/eLife.39648, PMID: 30444488 PMC6239433

[ref27] Cakin Memik N. Hunc F. Kalayci S. Demir N. Senturk E. Yildiz Gundogdu O.. (2023). Assessment of plasma-endogenous opioid neuropeptide levels and psychometric properties of non-suicidal self-injury in adolescents. Arch. Suicide Res. Off. J. Int. Acad. Suicide Res. 27, 749–768. doi: 10.1080/13811118.2022.2066494, PMID: 35499526

[ref28] Carter C. S. (2017). The oxytocin-vasopressin pathway in the context of love and fear. Front. Endocrinol. 8:356. doi: 10.3389/fendo.2017.00356, PMID: 29312146 PMC5743651

[ref29] Case A. Deaton A. (2020). Deaths of despair and the future of capitalism. Princeton, NJ: Princeton University Press.

[ref30] Castro D. C. Berridge K. C. (2014). Opioid hedonic hotspot in nucleus Accumbens Shell: mu, Delta, and kappa maps for enhancement of sweetness “liking” and “wanting. J. Neurosci. 34, 4239–4250. doi: 10.1523/JNEUROSCI.4458-13.2014, PMID: 24647944 PMC3960467

[ref31] Castro D. C. Bruchas M. R. (2019). A motivational and Neuropeptidergic hub: anatomical and functional diversity within the nucleus Accumbens Shell. Neuron 102, 529–552. doi: 10.1016/j.neuron.2019.03.003, PMID: 31071288 PMC6528838

[ref32] Chatterjee A. Vartanian O. (2014). Neuroaesthetics. Trends Cogn. Sci. 18, 370–375. doi: 10.1016/j.tics.2014.03.003, PMID: 24768244

[ref33] Chen P. Hong W. (2018). Neural circuit mechanisms of social behavior. Neuron 98, 16–30. doi: 10.1016/j.neuron.2018.02.026, PMID: 29621486 PMC6028944

[ref34] Chida Y. Steptoe A. (2009). The association of anger and hostility with future coronary heart disease: a meta-analytic review of prospective evidence. J. Am. Coll. Cardiol. 53, 936–946. doi: 10.1016/j.jacc.2008.11.044, PMID: 19281923

[ref35] Christie N. C. (2021). The role of social isolation in opioid addiction. Soc. Cogn. Affect. Neurosci. 16, 645–656. doi: 10.1093/scan/nsab029, PMID: 33681992 PMC8259283

[ref36] Chung E. Y. J. Oh J. S. (2022). Emotions in Korean philosophy and religion: Confucian, comparative, and contemporary perspectives [internet]. Cham: Springer International Publishing.

[ref37] Cinque C. Pondiki S. Oddi D. Di Certo M. G. Marinelli S. Troisi A.. (2012). Modeling socially anhedonic syndromes: genetic and pharmacological manipulation of opioid neurotransmission in mice. Transl. Psychiatry 2:e155. doi: 10.1038/tp.2012.83, PMID: 22929597 PMC3432195

[ref38] Coccaro E. F. Kavoussi R. J. Hauger R. L. Cooper T. B. Ferris C. F. (1998). Cerebrospinal fluid vasopressin levels: correlates with aggression and serotonin function in personality-disordered subjects. Arch. Gen. Psychiatry 55, 708–714. doi: 10.1001/archpsyc.55.8.708, PMID: 9707381

[ref39] Colonnello V. Iacobucci P. Fuchs T. Newberry R. C. Panksepp J. (2011). A useful animal model for social-affective neuroscience research: basic description of separation distress, social attachments and play. Neurosci. Biobehav. Rev. 35, 1854–1863. doi: 10.1016/j.neubiorev.2011.03.014, PMID: 21477615

[ref40] Couppis M. H. Kennedy C. H. (2008). The rewarding effect of aggression is reduced by nucleus accumbens dopamine receptor antagonism in mice. Psychopharmacology 197, 449–456. doi: 10.1007/s00213-007-1054-y, PMID: 18193405

[ref41] Csikszentmihalyi M. (1988). “The flow experience and its significance for human psychology” in Optimal experience: Psychological studies of flow in consciousness [internet]. eds. Csikszentmihalyi I. S. Csikszentmihalyi M. (Cambridge: Cambridge University Press), 15–35.

[ref42] Cumings B. (2005). Korea’s place in the sun: A modern history. Updated Edn. New York London: W.W. Norton & Company.

[ref43] Damsma G. Pfaus J. G. Wenkstern D. Phillips A. G. Fibiger H. C. (1992). Sexual behavior increases dopamine transmission in the nucleus accumbens and striatum of male rats: comparison with novelty and locomotion. Behav. Neurosci. 106, 181–191. doi: 10.1037/0735-7044.106.1.181, PMID: 1313243

[ref44] Darcq E. Kieffer B. L. (2018). Opioid receptors: drivers to addiction? Nat. Rev. Neurosci. 19, 499–514. doi: 10.1038/s41583-018-0028-x, PMID: 29934561

[ref45] Diamond J. M. (1999). Guns, germs, and steel: The fates of human societies. New York: Norton.

[ref46] Donahue R. J. Landino S. M. Golden S. A. Carroll F. I. Russo S. J. Carlezon W. A. (2015). Effects of acute and chronic social defeat stress are differentially mediated by the dynorphin/kappa-opioid receptor system. Behav. Pharmacol. 26, 654–663. doi: 10.1097/FBP.0000000000000155 26110224 PMC4586263

[ref47] Doo R. The Korea Herald. (2016). ‘Korea, a culture of desires’: Le Clezio. Available at: https://www.koreaherald.com/view.php?ud=20160602000755 (Accessed May 29, 2024).

[ref48] Dueñas M. Ojeda B. Salazar A. Mico J. A. Failde I. (2016). A review of chronic pain impact on patients, their social environment and the health care system. J. Pain Res. 9, 457–467. doi: 10.2147/JPR.S105892, PMID: 27418853 PMC4935027

[ref49] Dunbar R. I. M. (1998). The social brain hypothesis. Evol Anthropol Issues News Rev. 6, 178–190. doi: 10.1002/(SICI)1520-6505(1998)6:5<178::AID-EVAN5>3.0.CO;2-8, PMID: 39574662

[ref50] Dunbar R. I. M. Shultz S. (2010). Bondedness and sociality. Behaviour 147, 775–803. doi: 10.1163/000579510X501151

[ref51] Ebstein R. P. Israel S. Lerer E. Uzefovsky F. Shalev I. Gritsenko I.. (2009). Arginine vasopressin and oxytocin modulate human social behavior. Ann. N. Y. Acad. Sci. 1167, 87–102. doi: 10.1111/j.1749-6632.2009.04541.x 19580556

[ref177] Estiene C. (1972). Van Gogh: Critical Study. New York: Crown Publishers.

[ref52] Feng C. Hackett P. D. DeMarco A. C. Chen X. Stair S. Haroon E.. (2015). Oxytocin and vasopressin effects on the neural response to social cooperation are modulated by sex in humans. Brain Imaging Behav. 9, 754–764. doi: 10.1007/s11682-014-9333-9, PMID: 25416642

[ref53] Feng C. Qin L. Luo Y. Xu P. (2020). Intranasal vasopressin expedites dishonesty in women. Horm. Behav. 126:104843. doi: 10.1016/j.yhbeh.2020.104843, PMID: 32827501

[ref54] Filliol D. Ghozland S. Chluba J. Martin M. Matthes H. W. Simonin F.. (2000). Mice deficient for delta- and mu-opioid receptors exhibit opposing alterations of emotional responses. Nat. Genet. 25, 195–200. doi: 10.1038/76061, PMID: 10835636

[ref55] Gangestad S. W. Grebe N. M. (2017). Hormonal systems, human social bonding, and affiliation. Horm. Behav. 91, 122–135. doi: 10.1016/j.yhbeh.2016.08.005, PMID: 27530218

[ref56] Gerdes L. Tegeler C. H. Lee S. W. (2015). A groundwork for allostatic neuro-education. Front. Psychol. 6:1224. doi: 10.3389/fpsyg.2015.01224, PMID: 26347688 PMC4538224

[ref57] Gingrich B. Liu Y. Cascio C. Wang Z. Insel T. R. (2000). Dopamine D2 receptors in the nucleus accumbens are important for social attachment in female prairie voles (*Microtus ochrogaster*). Behav. Neurosci. 114, 173–183. doi: 10.1037/0735-7044.114.1.173, PMID: 10718272

[ref58] Golden S. A. Heins C. Venniro M. Caprioli D. Zhang M. Epstein D. H.. (2017). Compulsive addiction-like aggressive behavior in mice. Biol. Psychiatry 82, 239–248. doi: 10.1016/j.biopsych.2017.03.004, PMID: 28434654 PMC5532078

[ref59] Golden S. A. Jin M. Heins C. Venniro M. Michaelides M. Shaham Y. (2019). Nucleus Accumbens Drd1-expressing neurons control aggression self-administration and aggression seeking in mice. J. Neurosci. 39, 2482–2496. doi: 10.1523/JNEUROSCI.2409-18.2019, PMID: 30655356 PMC6435830

[ref60] Golden S. A. Shaham Y. (2018). Aggression addiction and relapse: a new frontier in psychiatry. Neuropsychopharmacol. Off. Publ. Am. Coll. Neuropsychopharmacol. 43, 224–225. doi: 10.1038/npp.2017.173, PMID: 29192664 PMC5719096

[ref61] Gosnell B. A. Levine A. S. Morley J. E. (1986). The stimulation of food intake by selective agonists of mu, kappa and delta opioid receptors. Life Sci. 38, 1081–1088. doi: 10.1016/0024-3205(86)90243-2, PMID: 2870405

[ref62] Graeber D. Wengrow D. (2021). The dawn of everything: A new history of humanity. First American Edn. New York: Farrar, Straus and Giroux. 10.1126/science.abm1652 34822293

[ref63] Guard H. J. Newman J. D. Roberts R. L. (2002). Morphine administration selectively facilitates social play in common marmosets. Dev. Psychobiol. 41, 37–49. doi: 10.1002/dev.10043, PMID: 12115289

[ref64] Gunderson J. G. Herpertz S. C. Skodol A. E. Torgersen S. Zanarini M. C. (2018). Borderline personality disorder. Nat. Rev. Dis. Primer 4:18029. doi: 10.1038/nrdp.2018.29 29795363

[ref65] Harlow H. F. Zimmermann R. R. (1959). Affectional responses in the infant monkey; orphaned baby monkeys develop a strong and persistent attachment to inanimate surrogate mothers. Science 130, 421–432. doi: 10.1126/science.130.3373.421, PMID: 13675765

[ref66] Harris E. (2023). Surgeon general offers strategy to tackle epidemic of loneliness. JAMA 329:1818. doi: 10.1001/jama.2023.8661, PMID: 37195692

[ref67] Hart S. (2023) Opinion|the wondrous connections between mathematics and literature. The New York Times. Available at: https://www.nytimes.com/2023/04/07/opinion/the-wondrous-connections-between-mathematics-and-literature.html (Accessed May 29, 2024).

[ref68] Henrich J. (2016). The secret of our success: How culture is driving human evolution, domesticating our species, and making us smarter. Princeton: Princeton University Press.

[ref69] Henrich J. P. (2020). The WEIRDest people in the world: How the west became psychologically peculiar and particularly prosperous. 1st Edn. New York: Farrar, Straus and Giroux.

[ref70] Henrich J. Heine S. J. Norenzayan A. (2010). The weirdest people in the world? Behav. Brain Sci. 33, 61–83. doi: 10.1017/S0140525X0999152X, PMID: 20550733

[ref71] Herman B. H. Panksepp J. (1978). Effects of morphine and naloxone on separation distress and approach attachment: evidence for opiate mediation of social affect. Pharmacol. Biochem. Behav. 9, 213–220. doi: 10.1016/0091-3057(78)90167-3, PMID: 568801

[ref72] Hinz L. D. Rim S. Lusebrink V. B. (2022). Clarifying the creative level of the expressive therapies continuum: A different dimension. Arts Psychother. 78:101896. doi: 10.1016/j.aip.2022.101896

[ref73] Holt-Lunstad J. (2022). Social connection as a public health issue: the evidence and a systemic framework for prioritizing the “social” in social determinants of health. Annu. Rev. Public Health 43, 193–213. doi: 10.1146/annurev-publhealth-052020-110732, PMID: 35021021

[ref74] Hsu D. T. Sanford B. J. Meyers K. K. Love T. M. Hazlett K. E. Walker S. J.. (2015). It still hurts: altered endogenous opioid activity in the brain during social rejection and acceptance in major depressive disorder. Mol. Psychiatry 20, 193–200. doi: 10.1038/mp.2014.185, PMID: 25600108 PMC4469367

[ref75] Hsu D. T. Sanford B. J. Meyers K. K. Love T. M. Hazlett K. E. Wang H.. (2013). Response of the μ-opioid system to social rejection and acceptance. Mol. Psychiatry 18, 1211–1217. doi: 10.1038/mp.2013.96, PMID: 23958960 PMC3814222

[ref76] Huang J. T. Reynolds S. D. DiGiovanni E. B. Zimmermann C. Joyce C. J. Katz J. T.. (2016). Fine arts curriculum improves observational skills of dermatology trainees: a pilot study. Br. J. Dermatol. 175, 815–817. doi: 10.1111/bjd.14616, PMID: 27037646

[ref77] Hui Gan G. Z. Hill A. M. Yeung P. Keesing S. Netto J. A. (2020). Pet ownership and its influence on mental health in older adults. Aging Ment. Health 24, 1605–1612. doi: 10.1080/13607863.2019.1633620, PMID: 31242754

[ref78] Hunt G. M. Azrin N. H. (1973). A community-reinforcement approach to alcoholism. Behav. Res. Ther. 11, 91–104. doi: 10.1016/0005-7967(73)90072-7, PMID: 4781962

[ref79] Iigaya K. O’Doherty J. P. Starr G. G. (2020). Progress and promise in Neuroaesthetics. Neuron 108, 594–596. doi: 10.1016/j.neuron.2020.10.022, PMID: 33242429

[ref80] Inagaki T. K. Hazlett L. I. Andreescu C. (2019). Naltrexone alters responses to social and physical warmth: implications for social bonding. Soc. Cogn. Affect. Neurosci. 14, 471–479. doi: 10.1093/scan/nsz026, PMID: 30976797 PMC6545530

[ref81] Inagaki T. K. Irwin M. R. Eisenberger N. I. (2015). Blocking opioids attenuates physical warmth-induced feelings of social connection. Emot. Wash DC. 15, 494–500. doi: 10.1037/emo0000088, PMID: 26098729 PMC4516568

[ref82] Inagaki T. K. Ray L. A. Irwin M. R. Way B. M. Eisenberger N. I. (2016). Opioids and social bonding: naltrexone reduces feelings of social connection. Soc. Cogn. Affect. Neurosci. 11, 728–735. doi: 10.1093/scan/nsw006, PMID: 26796966 PMC4847702

[ref83] Jackson M. Foret B. L. Fontenot J. Hasselschwert D. Smith J. Romero E.. (2023). Molecular examination of the endogenous opioid system in rhesus macaque monkeys with self-injurious behavior. J. Neurosci. Res. 101, 70–85. doi: 10.1002/jnr.25128, PMID: 36131680

[ref85] Johnson K. V. A. Dunbar R. I. M. (2016). Pain tolerance predicts human social network size. Sci. Rep. 6. doi: 10.1038/srep25267 PMC4848525 27121297

[ref86] Jurek B. Neumann I. D. (2018). The oxytocin receptor: from intracellular signaling to behavior. Physiol. Rev. 98, 1805–1908. doi: 10.1152/physrev.00031.2017, PMID: 29897293

[ref2] Kang M. (2022). “The problem with ‘han’ 한 恨”. Available at: https://aeon.co/essays/against-han-or-why-koreans-are-not-defined-by-sadness [accessed May 29, 2024].

[ref87] Kao H. T. Mürner-Lavanchy I. Lerch S. von Stosch E. Berger T. Koenig J.. (2024). Longitudinal associations between beta-endorphin, nonsuicidal self-injury and comorbid psychopathology. Psychiatry Res. 340:116142. doi: 10.1016/j.psychres.2024.116142, PMID: 39182317

[ref88] Kawada A. Nagasawa M. Murata A. Mogi K. Watanabe K. Kikusui T.. (2019). Vasopressin enhances human preemptive strike in both males and females. Sci. Rep. 9:9664. doi: 10.1038/s41598-019-45953-y, PMID: 31273244 PMC6609689

[ref89] Kennedy S. E. Koeppe R. A. Young E. A. Zubieta J. K. (2006). Dysregulation of endogenous opioid emotion regulation circuitry in major depression in women. Arch. Gen. Psychiatry 63, 1199–1208. doi: 10.1001/archpsyc.63.11.1199, PMID: 17088500

[ref90] Khosravi H. Khalilzadeh E. Vafaei S. G. (2021). Pain-induced aggression and changes in social behavior in mice. Aggress. Behav. 47, 89–98. doi: 10.1002/ab.21912, PMID: 32662216

[ref91] Kieffer B. L. Gavériaux-Ruff C. (2002). Exploring the opioid system by gene knockout. Prog. Neurobiol. 66, 285–306. doi: 10.1016/S0301-0082(02)00008-4, PMID: 12015197

[ref92] Kim I. J. Kim L. I. C. Kelly J. G. (2006). Developing cultural competence in working with Korean immigrant families. J. Community Psychol. 34, 149–165. doi: 10.1002/jcop.20093, PMID: 27013328

[ref93] Klaus M. H. Jerauld R. Kreger N. C. McAlpine W. Steffa M. Kennell J. H. (1972). Maternal attachment: importance of the first post-partum days. N. Engl. J. Med. 286, 460–463. doi: 10.1056/NEJM197203022860904, PMID: 5009748

[ref9001] Labarge E. (2024). “A New Perspective on Van Gogh’s Final Flowering.” New York Times, Available at: https://www.nytimes.com/2024/09/13/arts/design/van-gogh-poets-and-lovers-national-gallery-london.html (Accessed September 20, 2024).

[ref94] Le Merrer J. Becker J. A. J. Befort K. Kieffer B. L. (2009). Reward processing by the opioid system in the brain. Physiol. Rev. 89, 1379–1412. doi: 10.1152/physrev.00005.2009, PMID: 19789384 PMC4482114

[ref95] Lee S. W. (2019). A Copernican approach to brain advancement: the paradigm of allostatic orchestration. Front. Hum. Neurosci. 13:129. doi: 10.3389/fnhum.2019.00129, PMID: 31105539 PMC6499026

[ref96] Lee J. Wachholtz A. Choi K. H. (2014). A review of the Korean cultural syndrome Hwa-Byung: suggestions for theory and intervention. Asia Taepyongyang Sangdam Yongu 4:49. doi: 10.18401/2014.4.1.4, PMID: 25408922 PMC4232959

[ref97] Leichsenring F. Heim N. Leweke F. Spitzer C. Steinert C. Kernberg OF (2023). Borderline personality disorder: a review. JAMA 329, 670–679. doi: 10.1001/jama.2023.0589, PMID: 36853245

[ref98] Levis S. C. Mahler S. V. Baram T. Z. (2021). The developmental origins of opioid use disorder and its comorbidities. Front. Hum. Neurosci. 15:601905. doi: 10.3389/fnhum.2021.601905, PMID: 33643011 PMC7904686

[ref99] Leysen H. Walter D. Christiaenssen B. Vandoren R. Harputluoğlu İ. Van Loon N.. (2021). GPCRs are optimal regulators of complex biological systems and orchestrate the Interface between health and disease. Int. J. Mol. Sci. 22:13387. doi: 10.3390/ijms222413387, PMID: 34948182 PMC8708147

[ref100] Lightman S. L. Young W. S. (1987). Vasopressin, oxytocin, dynorphin, enkephalin and corticotrophin-releasing factor mRNA stimulation in the rat. J. Physiol. 394, 23–39. doi: 10.1113/jphysiol.1987.sp016858, PMID: 2895179 PMC1191949

[ref101] Lightman S. L. Young W. S. (1988). Corticotrophin-releasing factor, vasopressin and pro-opiomelanocortin mRNA responses to stress and opiates in the rat. J. Physiol. 403, 511–523. doi: 10.1113/jphysiol.1988.sp017261, PMID: 3267021 PMC1190725

[ref102] Lu L. Shepard J. D. Hall F. S. Shaham Y. (2003). Effect of environmental stressors on opiate and psychostimulant reinforcement, reinstatement and discrimination in rats: a review. Neurosci. Biobehav. Rev. 27, 457–491. doi: 10.1016/S0149-7634(03)00073-3, PMID: 14505687

[ref103] Lutz P. E. Kieffer B. L. (2013). The multiple facets of opioid receptor function: implications for addiction. Curr. Opin. Neurobiol. 23, 473–479. doi: 10.1016/j.conb.2013.02.005, PMID: 23453713 PMC3702666

[ref104] Machin A. J. Dunbar R. I. M. (2011). The brain opioid theory of social attachment: A review of the evidence. Behaviour 148, 985–1025. doi: 10.1163/000579511X596624

[ref105] Maher A. C. Kielb S. Loyer E. Connelley M. Rademaker A. Mesulam M. M.. (2017). Psychological well-being in elderly adults with extraordinary episodic memory. PLoS One 12:e0186413. doi: 10.1371/journal.pone.0186413, PMID: 29059208 PMC5653294

[ref106] Malone S. G. Shaykin J. D. Stairs D. J. Bardo M. T. (2022). Neurobehavioral effects of environmental enrichment and drug abuse vulnerability: an updated review. Pharmacol. Biochem. Behav. 221:173471. doi: 10.1016/j.pbb.2022.173471, PMID: 36228739 PMC10189610

[ref107] Mandavia A. Huang D. Wong J. Ruiz B. Crump F. Shen J.. (2017). Violating clan and kinship roles as risk factors for suicide and stigma among Lao refugees: an application of the cultural model of suicide and “what matters Most” frameworks. Isr. J. Psychiatry Relat. Sci. 54, 39–48, PMID: 28857757

[ref108] Manduca A. Lassalle O. Sepers M. Campolongo P. Cuomo V. Marsicano G.. (2016a). Interacting cannabinoid and opioid receptors in the nucleus Accumbens Core control adolescent social play. Front. Behav. Neurosci. 10:211. doi: 10.3389/fnbeh.2016.00211, PMID: 27899885 PMC5110529

[ref109] Manduca A. Servadio M. Damsteegt R. Campolongo P. Vanderschuren L. J. Trezza V. (2016b). Dopaminergic neurotransmission in the nucleus Accumbens modulates social play behavior in rats. Neuropsychopharmacol Off Publ Am Coll Neuropsychopharmacol. 41, 2215–2223. doi: 10.1038/npp.2016.22, PMID: 26860202 PMC4946055

[ref110] Manninen S. Tuominen L. Dunbar R. I. Karjalainen T. Hirvonen J. Arponen E.. (2017). Social laughter triggers endogenous opioid release in humans. J. Neurosci. 37, 6125–6131. doi: 10.1523/JNEUROSCI.0688-16.2017, PMID: 28536272 PMC6596504

[ref111] Marchant N. J. McDonald A. J. Matsuzaki R. van Mourik Y. Schetters D. De Vries T. J. (2023). Rats choose alcohol over social reward in an operant choice procedure. Neuropsychopharmacol. Off. Publ. Am. Coll. Neuropsychopharmacol. 48, 585–593. doi: 10.1038/s41386-022-01447-6, PMID: 36109596 PMC9938232

[ref112] Margari F. Lorusso M. Matera E. Pastore A. Zagaria G. Bruno F.. (2014). Aggression, impulsivity, and suicide risk in benign chronic pain patients - a cross-sectional study. Neuropsychiatr. Dis. Treat. 10, 1613–1620. doi: 10.2147/NDT.S66209, PMID: 25214787 PMC4159127

[ref113] Martin L. J. Hathaway G. Isbester K. Mirali S. Acland E. L. Niederstrasser N.. (2015). Reducing social stress elicits emotional contagion of pain in mouse and human strangers. Curr. Biol. CB 25, 326–332. doi: 10.1016/j.cub.2014.11.028, PMID: 25601547

[ref114] Martin L. J. Tuttle A. H. Mogil J. S. (2014). The interaction between pain and social behavior in humans and rodents. Curr. Top. Behav. Neurosci. 20, 233–250. doi: 10.1007/7854_2014_287 24557935

[ref115] Mason W. A. Saxon S. V. Sharpe L. G. (1963). Preferential responses of young chimpanzees to food and social rewards. Psychol. Rec. 13, 341–345. doi: 10.1007/BF03393535

[ref116] Massaccesi C. Willeit M. Quednow B. B. Nater U. M. Lamm C. Müller D.. (2022). Opioid-blunted cortisol response to stress is associated with increased negative mood and wanting of social reward. Neuropsychopharmacol. Off Publ. Am. Coll. Neuropsychopharmacol. 47, 1798–1807. doi: 10.1038/s41386-022-01283-8, PMID: 35140347 PMC9372154

[ref117] Masuda T. Batdorj B. Senzaki S. (2020). Culture and attention: future directions to expand research beyond the geographical regions of WEIRD cultures. Front. Psychol. 11:1394. doi: 10.3389/fpsyg.2020.01394, PMID: 32793021 PMC7393778

[ref118] Matthes H. W. Maldonado R. Simonin F. Valverde O. Slowe S. Kitchen I.. (1996). Loss of morphine-induced analgesia, reward effect and withdrawal symptoms in mice lacking the mu-opioid-receptor gene. Nature 383, 819–823. doi: 10.1038/383819a0, PMID: 8893006

[ref119] McEwen B. S. (2007). Physiology and neurobiology of stress and adaptation: central role of the brain. Physiol. Rev. 87, 873–904. doi: 10.1152/physrev.00041.2006, PMID: 17615391

[ref120] Mechling A. E. Arefin T. Lee H. L. Bienert T. Reisert M. Ben Hamida S.. (2016). Deletion of the mu opioid receptor gene in mice reshapes the reward-aversion connectome. Proc. Natl. Acad. Sci. USA 113, 11603–11608. doi: 10.1073/pnas.1601640113, PMID: 27671662 PMC5068324

[ref121] Meisel R. L. Joppa M. A. Rowe R. K. (1996). Dopamine receptor antagonists attenuate conditioned place preference following sexual behavior in female Syrian hamsters. Eur. J. Pharmacol. 309, 21–24. doi: 10.1016/0014-2999(96)00389-5, PMID: 8864688

[ref122] Milton J. (1667). Paradise Lost. Available at: https://www.gutenberg.org/files/26/26-h/26-h.htm and https://www.gutenberg.org/ebooks/26/pg26-images.html

[ref123] Min S. K. (2008). Clinical correlates of hwa-byung and a proposal for a new anger disorder. Psychiatry Investig. 5, 125–141. doi: 10.4306/pi.2008.5.3.125, PMID: 20046356 PMC2796026

[ref124] Moles A. Kieffer B. L. D’Amato F. R. (2004). Deficit in attachment behavior in mice lacking the mu-opioid receptor gene. Science 304, 1983–1986. doi: 10.1126/science.1095943, PMID: 15218152

[ref125] Murphy B. (2016). Van Gogh’s ear: The true story. First American Edn. New York: Farrar, Straus & Giroux, 319 p.

[ref126] Newman S. W. (1999). The medial extended amygdala in male reproductive behavior A node in the mammalian social behavior network. Ann. N. Y. Acad. Sci. 877, 242–257. doi: 10.1111/j.1749-6632.1999.tb09271.x, PMID: 10415653

[ref127] Newman E. L. Covington H. E. Suh J. Bicakci M. B. Ressler K. J. DeBold J. F.. (2019). Fighting females: neural and behavioral consequences of social defeat stress in female mice. Biol. Psychiatry 86, 657–668. doi: 10.1016/j.biopsych.2019.05.005, PMID: 31255250 PMC6788975

[ref128] Nisbett R. E. (2004). The geography of thought: how Asians and westerners think differently… And why. 1. Free press trade paperback. New York: Free Press.

[ref129] Noguchi T. Ikeda T. Kanai T. Saito M. Kondo K. Saito T. (2023). Association of social isolation and loneliness with chronic low back pain among older adults: A cross-sectional study from Japan Gerontological evaluation study (JAGES). J. Epidemiol. 34, 270–277. doi: 10.2188/jea.JE20230127 PMC11078594 37690817

[ref130] Normansell L. Panksepp J. (1990). Effects of morphine and naloxone on play-rewarded spatial discrimination in juvenile rats. Dev. Psychobiol. 23, 75–83. doi: 10.1002/dev.420230108, PMID: 2160387

[ref131] Nummenmaa L. Karjalainen T. Isojärvi J. Kantonen T. Tuisku J. Kaasinen V.. (2020). Lowered endogenous mu-opioid receptor availability in subclinical depression and anxiety. Neuropsychopharmacol. Off Publ. Am. Coll. Neuropsychopharmacol. 45, 1953–1959. doi: 10.1038/s41386-020-0725-9, PMID: 32473595 PMC7608336

[ref132] Nummenmaa L. Manninen S. Tuominen L. Hirvonen J. Kalliokoski K. K. Nuutila P.. (2015). Adult attachment style is associated with cerebral μ-opioid receptor availability in humans. Hum. Brain Mapp. 36, 3621–3628. doi: 10.1002/hbm.22866, PMID: 26046928 PMC6869236

[ref133] Oddi D. Crusio W. E. D’Amato F. R. Pietropaolo S. (2013). Monogenic mouse models of social dysfunction: implications for autism. Behav. Brain Res. 251, 75–84. doi: 10.1016/j.bbr.2013.01.002, PMID: 23327738

[ref134] Oliva A. Torralba A. (2007). The role of context in object recognition. Trends Cogn. Sci. 11, 520–527. doi: 10.1016/j.tics.2007.09.009, PMID: 18024143

[ref135] Oliveira Ve De M. Lukas M. Wolf H. N. Durante E. Lorenz A. Mayer A. L.. (2021). Oxytocin and vasopressin within the ventral and dorsal lateral septum modulate aggression in female rats. Nat. Commun. 12. doi: 10.1038/s41467-021-23064-5 PMC8131389 34006875

[ref136] Oliveira Ve De M. Neumann I. D. De Jong T. R. (2019). Post-weaning social isolation exacerbates aggression in both sexes and affects the vasopressin and oxytocin system in a sex-specific manner. Neuropharmacology 156:107504. doi: 10.1016/j.neuropharm.2019.01.019 30664846

[ref137] Pandey V. Yadav V. Singh R. Srivastava A. Subhashini. (2023). β-endorphin (an endogenous opioid) inhibits inflammation, oxidative stress and apoptosis via Nrf-2 in asthmatic murine model. Sci. Rep. 13. doi: 10.1038/s41598-023-38366-5 PMC10390559 37524754

[ref138] Panksepp J. Bean N. J. Bishop P. Vilberg T. Sahley T. L. (1980). Opioid blockade and social comfort in chicks. Pharmacol. Biochem. Behav. 13, 673–683. doi: 10.1016/0091-3057(80)90011-8, PMID: 7443737

[ref139] Panksepp J. Herman B. H. Vilberg T. Bishop P. DeEskinazi F. G. (1980). Endogenous opioids and social behavior. Neurosci. Biobehav. Rev. 4, 473–487. doi: 10.1016/0149-7634(80)90036-6, PMID: 6258111

[ref140] Park I. (2022). “Korean social emotions: Han (한 恨), Heung (흥 興), and Jeong (정 情)” in Emotions in Korean philosophy and religion: Confucian, comparative, and contemporary perspectives. eds. Chung E. Y. J. Sophia Oh J. (Cham: Springer International Publishing).

[ref141] Pellissier L. P. Gandía J. Laboute T. Becker J. A. J. Le Merrer J. (2018). μ opioid receptor, social behaviour and autism spectrum disorder: reward matters. Br. J. Pharmacol. 175, 2750–2769. doi: 10.1111/bph.13808, PMID: 28369738 PMC6016638

[ref142] Peloquin S. M. (1996). Art: an occupation with promise for developing empathy. Am. J. Occup. Ther. Off. Publ. Am. Occup. Ther. Assoc. 50, 655–661. doi: 10.5014/ajot.50.8.655, PMID: 8863938

[ref143] Peng Y. Hahn R. A. Finnie R. K. C. Cobb J. Williams S. P. Fielding J. E.. (2020). Permanent supportive housing with housing first to reduce homelessness and promote health among homeless populations with disability: A community guide systematic review. J. Public Health Manag. Pract. 26, 404–411. doi: 10.1097/PHH.0000000000001219, PMID: 32732712 PMC8513528

[ref144] Perez-Rodriguez M. M. Bulbena-Cabré A. Bassir Nia A. Zipursky G. Goodman M. New A. S. (2018). The neurobiology of borderline personality disorder. Psychiatr. Clin. North Am. 41, 633–650. doi: 10.1016/j.psc.2018.07.012, PMID: 30447729

[ref145] Pfaff D. W. Joels M. (2016). Hormones, brain and behavior. 3rd Edn. Oxford, United Kingdom: Elsevier.

[ref146] Phelps S. M. Okhovat M. Berrio A. (2017). Individual differences in social behavior and cortical vasopressin receptor: genetics, epigenetics, and evolution. Front. Neurosci. 11:537. doi: 10.3389/fnins.2017.00537, PMID: 29085274 PMC5649215

[ref147] Pilozzi A. Carro C. Huang X. (2020). Roles of β-endorphin in stress, behavior, Neuroinflammation, and brain energy metabolism. Int. J. Mol. Sci. 22:338. doi: 10.3390/ijms22010338, PMID: 33396962 PMC7796446

[ref148] Poulain M. Herm A. Errigo A. Chrysohoou C. Legrand R. Passarino G.. (2021). Specific features of the oldest old from the longevity blue zones in Ikaria and Sardinia. Mech. Ageing Dev. 198:111543. doi: 10.1016/j.mad.2021.111543, PMID: 34265327

[ref149] Poulain M. Herm A. Pes G. (2013). The blue zones: areas of exceptional longevity around the world. Vienna Yearb Popul Res. 11, 87–108. doi: 10.1553/populationyearbook2013s87

[ref150] Prezzano S. C. (1997). “Warfare, women, and households: the development of Iroquois culture” in Women and prehistory: North America and Mesoamerica (Philadelphia: University of Pennsylvania Press).

[ref151] Prossin A. R. Love T. M. Koeppe R. A. Zubieta J. K. Silk K. R. (2010). Dysregulation of regional endogenous opioid function in borderline personality disorder. Am. J. Psychiatry 167, 925–933. doi: 10.1176/appi.ajp.2010.09091348, PMID: 20439388 PMC6863154

[ref152] Ragnauth A. Moroz M. Bodnar R. J. (2000). Multiple opioid receptors mediate feeding elicited by mu and delta opioid receptor subtype agonists in the nucleus accumbens shell in rats. Brain Res. 876, 76–87. doi: 10.1016/S0006-8993(00)02631-7, PMID: 10973595

[ref153] Ren W. Centeno M. V. Wei X. Wickersham I. Martina M. Apkarian A. V.. (2021). Adaptive alterations in the mesoaccumbal network after peripheral nerve injury. Pain 162, 895–906. doi: 10.1097/j.pain.0000000000002092, PMID: 33021562 PMC9272541

[ref154] Renaud S. de Lorgeril M. (1992). Wine, alcohol, platelets, and the French paradox for coronary heart disease. Lancet Lond Engl. 339, 1523–1526. doi: 10.1016/0140-6736(92)91277-F, PMID: 1351198

[ref155] Richard J. M. Fields H. L. (2016). Mu-opioid receptor activation in the medial shell of nucleus accumbens promotes alcohol consumption, self-administration and cue-induced reinstatement. Neuropharmacology 108, 14–23. doi: 10.1016/j.neuropharm.2016.04.010, PMID: 27089981 PMC4912898

[ref156] Rigney N. de Vries G. J. Petrulis A. (2023). Modulation of social behavior by distinct vasopressin sources. Front. Endocrinol. 14:1127792. doi: 10.3389/fendo.2023.1127792, PMID: 36860367 PMC9968743

[ref157] Rosen D. Oh Y. Chesebrough C. Zhang F. Z. Kounios J. (2024). Creative flow as optimized processing: evidence from brain oscillations during jazz improvisations by expert and non-expert musicians. Neuropsychologia 196:108824. doi: 10.1016/j.neuropsychologia.2024.108824, PMID: 38387554

[ref158] Runyan W. M. (1981). Why did Van Gogh cut off his ear? The problem of alternative explanations in psychobiography. J. Pers. Soc. Psychol. 40, 1070–1077. doi: 10.1037/0022-3514.40.6.1070, PMID: 7021798

[ref9002] Russell B. (2004). History of Western Philosophy. New York: Routledge.

[ref159] Russell J. A. Neumann I. Landgraf R. (1992). Oxytocin and vasopressin release in discrete brain areas after naloxone in morphine-tolerant and -dependent anesthetized rats: push-pull perfusion study. J. Neurosci. 12, 1024–1032. doi: 10.1523/JNEUROSCI.12-03-01024.1992, PMID: 1545232 PMC6576032

[ref160] Schedlowski M. Enck P. Rief W. Bingel U. (2015). Neuro-bio-behavioral mechanisms of placebo and nocebo responses: implications for clinical trials and clinical practice. Pharmacol. Rev. 67, 697–730. doi: 10.1124/pr.114.009423, PMID: 26126649

[ref161] Schmahl C. Bremner J. D. (2006). Neuroimaging in borderline personality disorder. J. Psychiatr. Res. 40, 419–427. doi: 10.1016/j.jpsychires.2005.08.011, PMID: 16239012 PMC3233768

[ref162] Shah C. R. Forsberg C. G. Kang J. Q. Veenstra-VanderWeele J. (2013). Letting a typical mouse judge whether mouse social interactions are atypical. Autism Res. Off. J. Int. Soc. Autism Res. 6, 212–220. doi: 10.1002/aur.1280, PMID: 23436806 PMC3664268

[ref163] Smagin D. A. Galyamina A. G. Kovalenko I. L. Kudryavtseva N. N. (2022). Altered expression of genes associated with major neurotransmitter Systems in the Reward-Related Brain Regions of mice with positive fighting experience. Int. J. Mol. Sci. 23:13644. doi: 10.3390/ijms232113644, PMID: 36362437 PMC9655062

[ref164] Stanley B. Siever L. J. (2010). The interpersonal dimension of borderline personality disorder: toward a neuropeptide model. Am. J. Psychiatry 167, 24–39. doi: 10.1176/appi.ajp.2009.09050744, PMID: 19952075 PMC4176877

[ref165] Stein D. J. van Honk J. Ipser J. Solms M. Panksepp J. (2007). Opioids: from physical pain to the pain of social isolation. CNS Spectr. 12, 669–674. doi: 10.1017/S1092852900021490, PMID: 17805212

[ref166] Störkel L. M. Karabatsiakis A. Hepp J. Kolassa I. T. Schmahl C. Niedtfeld I. (2021). Salivary beta-endorphin in nonsuicidal self-injury: an ambulatory assessment study. Neuropsychopharmacol. Off. Publ. Am. Coll. Neuropsychopharmacol. 46, 1357–1363. doi: 10.1038/s41386-020-00914-2, PMID: 33398083 PMC8134499

[ref167] Sullivan M. D. Ballantyne J. C. (2021). When physical and social pain coexist: insights into opioid therapy. Ann. Fam. Med. 19, 79–82. doi: 10.1370/afm.2591, PMID: 33355099 PMC7800754

[ref168] Suzuki Y. Suzuki T. Takagi M. Murakami M. Ikeda T. (2024). Bidirectional longitudinal association between Back pain and loneliness in later life: evidence from English longitudinal study of ageing. Ann Geriatr Med Res. 28, 27–35. doi: 10.4235/agmr.23.0136, PMID: 38105012 PMC10982446

[ref169] Terranova J. I. Ferris C. F. Albers H. E. (2017). Sex differences in the regulation of offensive aggression and dominance by arginine-vasopressin. Front. Endocrinol. 8:308. doi: 10.3389/fendo.2017.00308, PMID: 29184535 PMC5694440

[ref170] Terranova J. I. Song Z. Larkin T. E. Hardcastle N. Norvelle A. Riaz A.. (2016). Serotonin and arginine-vasopressin mediate sex differences in the regulation of dominance and aggression by the social brain. Proc. Natl. Acad. Sci. USA 113, 13233–13238. doi: 10.1073/pnas.1610446113, PMID: 27807133 PMC5135349

[ref171] Thompson R. R. George K. Walton J. C. Orr S. P. Benson J. (2006). Sex-specific influences of vasopressin on human social communication. Proc. Natl. Acad. Sci. USA 103, 7889–7894. doi: 10.1073/pnas.0600406103, PMID: 16682649 PMC1472540

[ref172] Timm A. Schmidt-Wilcke T. Blenk S. Studer B. (2023). Altered social decision making in patients with chronic pain. Psychol. Med. 53, 2466–2475. doi: 10.1017/S0033291721004359, PMID: 34736548 PMC10123842

[ref173] Toddes C. Lefevre E. M. Brandner D. D. Zugschwert L. Rothwell P. E. (2021). μ-Opioid receptor (Oprm1) copy number influences nucleus accumbens microcircuitry and reciprocal social behaviors. J. Neurosci. 41, 7965–7977. doi: 10.1523/JNEUROSCI.2440-20.2021, PMID: 34301826 PMC8460143

[ref174] Trezza V. Baarendse P. J. J. Vanderschuren L. J. M. J. (2010). The pleasures of play: pharmacological insights into social reward mechanisms. Trends Pharmacol. Sci. 31, 463–469. doi: 10.1016/j.tips.2010.06.008, PMID: 20684996 PMC2946511

[ref175] Troisi A. Frazzetto G. Carola V. Di Lorenzo G. Coviello M. D’Amato F. R.. (2011). Social hedonic capacity is associated with the A118G polymorphism of the mu-opioid receptor gene (OPRM1) in adult healthy volunteers and psychiatric patients. Soc. Neurosci. 6, 88–97. doi: 10.1080/17470919.2010.482786, PMID: 20486014

[ref176] van der Venne P. Balint A. Drews E. Parzer P. Resch F. Koenig J.. (2021). Pain sensitivity and plasma beta-endorphin in adolescent non-suicidal self-injury. J. Affect. Disord. 278, 199–208. doi: 10.1016/j.jad.2020.09.036, PMID: 32961416

[ref178] Van Gogh’s ear/the pact of silence. (2008). Available at: https://vangoghsear.com/index.html (Accessed May 29, 2024).

[ref179] van Londen L. Goekoop J. G. van Kempen G. M. Frankhuijzen-Sierevogel A. C. Wiegant V. M. van der Velde E. A.. (1997). Plasma levels of arginine vasopressin elevated in patients with major depression. Neuropsychopharmacol. Off Publ. Am. Coll. Neuropsychopharmacol. 17, 284–292. doi: 10.1016/S0893-133X(97)00054-7, PMID: 9326754

[ref180] Vanderschuren L. J. M. J. Achterberg E. J. M. Trezza V. (2016). The neurobiology of social play and its rewarding value in rats. Neurosci. Biobehav. Rev. 70, 86–105. doi: 10.1016/j.neubiorev.2016.07.025, PMID: 27587003 PMC5074863

[ref181] Vanderschuren L. J. Niesink R. J. Spruijt B. M. Van Ree J. M. (1995). Effects of morphine on different aspects of social play in juvenile rats. Psychopharmacology 117, 225–231. doi: 10.1007/BF02245191, PMID: 7753971

[ref182] Venniro M. Marino R. A. M. Chow J. J. Caprioli D. Epstein D. H. Ramsey L. A.. (2022). The protective effect of social reward on opioid and psychostimulant reward and relapse: behavior, pharmacology, and brain regions. J. Neurosci. 42, 9298–9314. doi: 10.1523/JNEUROSCI.0931-22.2022, PMID: 36517252 PMC9794371

[ref183] Venniro M. Panlilio L. V. Epstein D. H. Shaham Y. (2021). The protective effect of operant social reward on cocaine self-administration, choice, and relapse is dependent on delay and effort for the social reward. Neuropsychopharmacol. Off. Publ. Am. Coll. Neuropsychopharmacol. 46, 2350–2357. doi: 10.1038/s41386-021-01148-6, PMID: 34400784 PMC8580997

[ref184] Venniro M. Russell T. I. Zhang M. Shaham Y. (2019). Operant social reward decreases incubation of heroin craving in male and female rats. Biol. Psychiatry 86, 848–856. doi: 10.1016/j.biopsych.2019.05.018, PMID: 31326085 PMC8383184

[ref185] Venniro M. Zhang M. Caprioli D. Hoots J. K. Golden S. A. Heins C.. (2018). Volitional social interaction prevents drug addiction in rat models. Nat. Neurosci. 21, 1520–1529. doi: 10.1038/s41593-018-0246-6, PMID: 30323276 PMC7386559

[ref186] Vivian J. A. Miczek K. A. (1999). Interactions between social stress and morphine in the periaqueductal gray: effects on affective vocal and reflexive pain responses in rats. Psychopharmacology 146, 153–161. doi: 10.1007/s002130051101, PMID: 10525750

[ref9003] Wallace R. (1969). The World of Van Gogh: 1853-1890. New York: Time-Life Books, p.95.

[ref187] Way B. M. Taylor S. E. Eisenberger N. I. (2009). Variation in the mu-opioid receptor gene (OPRM1) is associated with dispositional and neural sensitivity to social rejection. Proc. Natl. Acad. Sci. USA 106, 15079–15084. doi: 10.1073/pnas.0812612106, PMID: 19706472 PMC2736434

[ref84] Weisstuch L. (2023). Jobless, divorced, on probation; a pandemic hobby turned his life around. The New York Times. Available at: https://www.nytimes.com/2023/09/08/nyregion/ice-box-model-nyc.html (Accessed May 29, 2024).

[ref188] Yan R. Wei D. Varshneya A. Shan L. Dai B. Asencio H. J.. (2024). The multi-stage plasticity in the aggression circuit underlying the winner effect. Cell. 187, 6785.–6803.e18. doi: 10.1016/j.cell.2024.09.030 39406242 PMC11784869

[ref189] Yang T. C. Shoff C. Kim S. (1982a). Social isolation, residential stability, and opioid use disorder among older Medicare beneficiaries: metropolitan and non-metropolitan county comparison. Soc. Sci. Med. 292:114605. doi: 10.1016/j.socscimed.2021.114605 PMC8748391 34861571

[ref190] Yang T. C. Shoff C. Kim S. Shaw B. A. (1982b). County social isolation and opioid use disorder among older adults: A longitudinal analysis of Medicare data, 2013-2018. Soc. Sci. Med. 301:114971. doi: 10.1016/j.socscimed.2022.114971 PMC10211474 35430465

[ref191] Zhao B. G. Chapman C. Bicknell R. J. (1988). Opioid-noradrenergic interactions in the neurohypophysis. I. Differential opioid receptor regulation of oxytocin, vasopressin, and noradrenaline release. Neuroendocrinology 48, 16–24. doi: 10.1159/000124984, PMID: 2845291

[ref192] Zheng X. Zhang T. Ding H. Wang C. (1995). Plasma levels of beta-endorphin, leucine enkephalin and arginine vasopressin in patients with essential hypertension and the effects of clonidine. Int. J. Cardiol. 51, 233–244. doi: 10.1016/0167-5273(95)02423-T, PMID: 8586472

[ref193] Zubieta J. K. Bueller J. A. Jackson L. R. Scott D. J. Xu Y. Koeppe R. A.. (2005). Placebo effects mediated by endogenous opioid activity on mu-opioid receptors. J. Neurosci. 25, 7754–7762. doi: 10.1523/JNEUROSCI.0439-05.2005, PMID: 16120776 PMC6725254

[ref194] Room/International Institute of Psychology. Available at: http://www.nlphy.com/bbs/board.php?bo_table=b07&wr_id=839 (Accessed May 29, 2024).

